# Multi-omic Directed Discovery of Cellulosomes, Polysaccharide Utilization Loci, and Lignocellulases from an Enriched Rumen Anaerobic Consortium

**DOI:** 10.1128/AEM.00199-20

**Published:** 2020-09-01

**Authors:** Geizecler Tomazetto, Agnes C. Pimentel, Daniel Wibberg, Neil Dixon, Fabio M. Squina

**Affiliations:** a Programa de Processos Tecnológicos e Ambientais, Universidade de Sorocaba, Sorocaba, Brazil; b Manchester Institute of Biotechnology, Department of Chemistry, University of Manchester, Manchester, United Kingdom; c Departamento de Bioquímica, Instituto de Biologia (IB), Universidade Estadual de Campinas (UNICAMP), Cidade Universitária, Campinas, São Paulo, Brazil; d Center for Biotechnology (CeBiTec), Genome Research of Industrial Microorganisms, Bielefeld University, Bielefeld, Germany; INRS—Institut Armand-Frappier

**Keywords:** anaerobic consortium, lignocellulose degradation, metagenome, metasecretome, polysaccharide utilization loci, rumen

## Abstract

Lignocellulose is one of the most abundant renewable carbon sources, representing an alternative to petroleum for the production of fuel and chemicals. Nonetheless, the lignocellulose saccharification process, to release sugars for downstream applications, is one of the most crucial factors economically challenging to its use. The synergism required among the various carbohydrate-active enzymes (CAZymes) for efficient lignocellulose breakdown is often not satisfactorily achieved with an enzyme mixture from a single strain. To overcome this challenge, enrichment strategies can be applied to develop microbial communities with an efficient CAZyme arsenal, incorporating complementary and synergistic properties, to improve lignocellulose deconstruction. We report a comprehensive and deep analysis of an enriched rumen anaerobic consortium (ERAC) established on sugarcane bagasse (SB). The lignocellulolytic abilities of the ERAC were confirmed by analyzing the depolymerization of bagasse by scanning electron microscopy, enzymatic assays, and mass spectrometry. Taxonomic analysis based on 16S rRNA sequencing elucidated the community enrichment process, which was marked by a higher abundance of *Firmicutes* and *Synergistetes* species. Shotgun metagenomic sequencing of the ERAC disclosed 41 metagenome-assembled genomes (MAGs) harboring cellulosomes and polysaccharide utilization loci (PULs), along with a high diversity of CAZymes. The amino acid sequences of the majority of the predicted CAZymes (60% of the total) shared less than 90% identity with the sequences found in public databases. Additionally, a clostridial MAG identified in this study produced proteins during consortium development with scaffoldin domains and CAZymes appended to dockerin modules, thus representing a novel cellulosome-producing microorganism.

**IMPORTANCE** The lignocellulolytic ERAC displays a unique set of plant polysaccharide-degrading enzymes (with multimodular characteristics), cellulosomal complexes, and PULs. The MAGs described here represent an expansion of the genetic content of rumen bacterial genomes dedicated to plant polysaccharide degradation, therefore providing a valuable resource for the development of biocatalytic toolbox strategies to be applied to lignocellulose-based biorefineries.

## Abstract (precis)

The lignocellulolytic ERAC displays a unique set of plant polysaccharide-degrading enzymes (with multimodular characteristics), cellulosomal complexes, and PULs. The MAGs described here represent an expansion of the genetic content of rumen bacterial genomes dedicated to plant polysaccharide degradation, therefore providing a valuable resource for the development of biocatalytic toolbox strategies to be applied to lignocellulose-based biorefineries.

## INTRODUCTION

Lignocellulosic biomass represents the most abundant source of renewable carbon. It is an attractive and sustainable alternative to petroleum for the production of biofuels, chemicals, and other biomaterials (1). For example, large amounts of lignocellulosic residues generated in biorefineries, such as sugarcane bagasse (SB) in bioethanol production plants, could be employed as raw material, instead of being used in boilers as an energy supply (2–4). Lignocellulosic biomass is composed of cellulose, hemicellulose, and lignin, which are highly organized and interlinked by a variety of covalent bonds, forming a recalcitrant structure. Therefore, the bioconversion of lignocellulosic polymers into bioproducts requires an enzymatic cocktail capable of acting on the different bonds of the substrate (2).

In nature, biomass is efficiently degraded by microbial communities present in different ecosystems, such as soil (5), rumen (6–8), and insect gut (9). Overall, the microbial communities are composed of taxonomically different microorganisms capable of secreting a large array of enzymes with different substrate specificities. Among these ecosystems, the rumen microbiome is composed of a highly diverse and complex mixture of bacteria, archaea, fungi, and protozoa with a remarkable ability to break down a variety of biomasses (6, 8, 10, 11). This microbiome represents a promising reservoir of enzymes for applications in lignocellulose-based biorefineries (6, 8, 12).

The genomes of rumen microorganisms encode a broad selection of multifunctional carbohydrate-active enzymes (CAZymes), which typically contain a catalytic domain and one or more noncatalytic domains, which include carbohydrate-binding modules (CBM), dockerins, and fibronectin 3-like modules (6, 7, 12, 13). In this biological system, microbial taxa can assemble their CAZymes in multimodular enzymatic complexes. For example, *Clostridium* species can organize a multifunctional enzymatic system (with different catalytic domains) onto a scaffoldin protein, which is attached to the cell surface (14). These multifunctional complexes found in Clostridium thermocellum and Ruminococcus flavefaciens are termed “cellulosomes” (15).

Some *Bacteroidetes* bacteria possess gene clusters that depolymerize glycans, and these are called polysaccharide utilization loci (PULs) (16, 17). The PULs are gene clusters encoding CAZymes, surface glycan-binding proteins, oligosaccharides transporters, or transcriptional regulators (17). In this system, the bacteria secrete PUL-associated CAZymes that degrade polysaccharides into oligomers, which are transported to the periplasm by transporters encoded by *susCD*-like genes for complete degradation (16, 17).

Several studies based on culture-dependent and -independent methods have uncovered the CAZyme repertoires of rumen anaerobic species, depicting their strategies for lignocellulosic biomass digestion (6, 8, 12, 18). Based on a culture-dependent approach, the Hungate1000 project recently presented the CAZyme profiles of more than 400 bacterial and archaeal genomes of microbial isolates from rumen samples (12). Using culture-independent methods, an ultradeep metagenomic sequencing from 283 cattle samples revealed the CAZyme repertoire of 4,941 rumen uncultured genomes (RUGs) (8). Such genome-centric metagenomic approaches provide more detail that helps provide an understanding of the phylogenetic and metabolic properties of individual genomes, allowing one to propose novel candidate species and comprehension of the syntrophic interactions among members of microbial communities (19–21). By combining metagenomic and metaproteome analyses, it is possible to depict the key enzymes produced during consortium development under precise conditions, rather than just identify the genetic information of the microbial community (22). Independently of the approach applied, these studies consistently report that the rumen microbiome remains a rich and untapped source of new CAZymes and multienzymatic complexes (6–8, 12, 13, 23).

A powerful strategy to disclose enzymatic complexes of relevance for biorefinery-related applications is based on enrichment strategies (23–27). The enrichment forces shifts in the diversity of microbial communities in response to specific carbon source (28–31). This strategy is not inoculum driven (28) and allows the enrichment of microbial genes related to a specific metabolism (23). A recent study of microbial consortia developed from beaver and moose rumen gut microbiota described the resulting microbial composition, which responded differently to each one of the four lignocellulosic carbon sources used during the enrichment processes (28).

In this study, we established an enriched rumen anaerobic consortium (ERAC), enriched for several weeks, using sugarcane bagasse and rumen as unique carbon and microbial sources, respectively. To investigate whether the recalcitrance of the plant biomass selects for promising degrading microorganisms from the rumen endowed with diverse CAZymes and able to induce the production of natural enzymatic cocktails, a multi-omics discovery strategy was applied. The taxonomic analysis, based on bacterial ribosomal gene sequencing, showed the enrichment of phylogenetic groups, known as polysaccharides degraders, such as *Firmicutes* and *Synergistetes*. A metagenomic approach allowed the reconstruction of several metagenome assembly genomes (MAGs), as well as the identification of an extensive repertoire of genes encoding CAZymes, and their protein products were confirmed by metaproteomic analysis. The lignocellulolytic abilities of the anaerobic consortium in the deconstruction of bagasse were further confirmed by scanning electron microscopy (SEM), enzymatic assays, and assessment of the metabolic activity consortium by measurement of the gases produced.

## RESULTS

### Lignocellulolytic evaluation of an ERAC.

**FIG 1:**
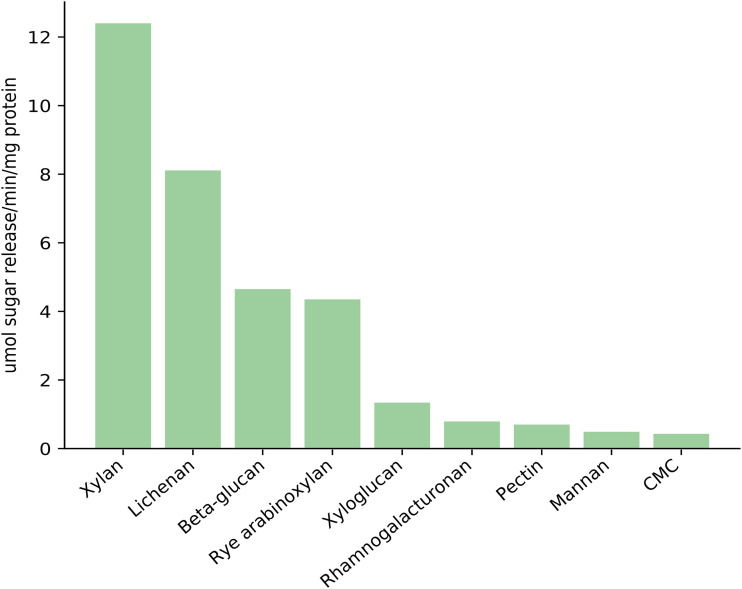
Biochemical assays using the enriched rumen anaerobic consortium (ERAC) metaproteome against nine different substrates. Reducing sugars were released from reactions of the ERAC metaproteome against xylan, lichenan, β-glucan, rye arabinoxylan, xyloglucan, rhamnogalacturonan, pectin, mannan, and carboxymethyl cellulose sodium salt (CMC).

An enriched rumen anaerobic consortium (ERAC) was established using a rumen sample as an inoculum, which was then subjected to 25 sequential transfers into fresh medium every 5 days under anaerobic conditions. The detection of carbon dioxide (CO_2_) and hydrogen (H_2_) by gas chromatography (GC)-mass spectrometry (MS) confirmed the anaerobic metabolism of the ERAC (see Table S1 in the supplemental material). As described in Fig. 1, the culture medium supernatant presented the ability to break down natural polysaccharides. The enzymatic assays were performed against nine distinct polysaccharides, with the greatest activity being observed against xylan, lichenan, β-glucan, and rye arabinoxylan, confirming that the consortium was able to produce an array of enzymes for cellulose and hemicellulose degradation.

**FIG 2:**
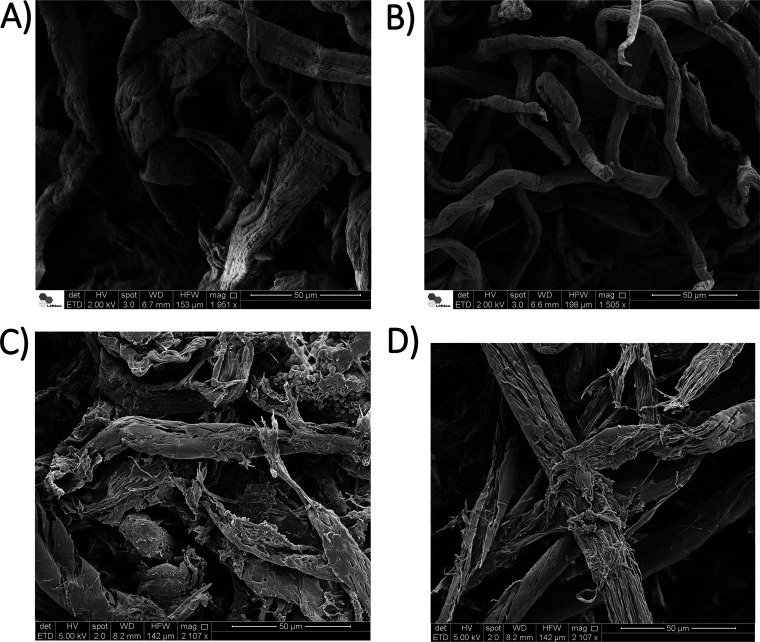
Scanning electron microscopy images of the sugarcane bagasse prior to incubation (A and B) and after 7 days of incubation (C and D) with the enriched rumen anaerobic consortium (ERAC).

We examined by SEM whether the ERAC could cause modifications to sugarcane bagasse. Several SEM images of the bagasse samples were obtained prior to and after 7 days of incubation with ERAC (Fig. 2). The sugarcane bagasse control (no incubation) showed fibers with a continuous surface (Fig. 2A and B), whereas clear visual signs of decomposition were observed in the bagasse fibers following incubation with ERAC (Fig. 2C and D). Collectively, these functional data confirmed the *ex situ* enrichment of a rumen-derived anaerobic consortium able to break down sugarcane bagasse.

### Impact of enrichment on taxonomic profile and diversity indices.

The impact of enrichment of the cow rumen-derived inoculum sample in response to sugarcane bagasse on the microbial structure, richness, and diversity was determined and calculated based on the 16S rRNA amplicon sequences. High-throughput sequencing yielded 322,680 and 281,340 high-quality sequences for the original cow rumen and ERAC samples, respectively (Table S2). Clustering of these partial 16S rRNA gene sequences resulted in 721 and 312 species-level operational taxonomic units (OTUs) for the cow rumen and ERAC, respectively, indicating a decrease in the biodiversity within the enriched culture. Consistent with this interpretation, richness (ACE and Chao1) and diversity (Shannon and Simpson) indices were lower for ERAC than for cow rumen (Table S3). Moreover, the rarefaction curves reached a plateau in both cases (Fig. S1), suggesting that the microbial communities were entirely covered, permitting a robust estimate of bacterial species richness and diversity.

**FIG 3:**
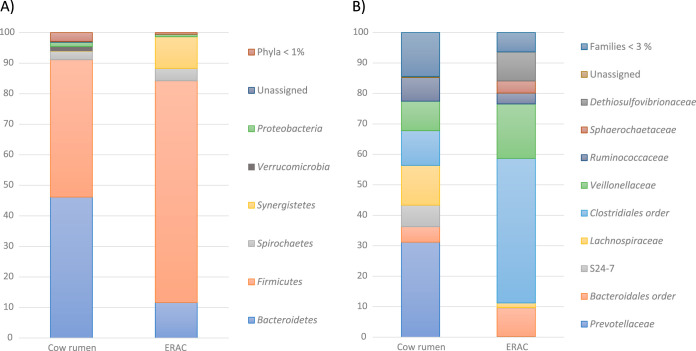
Relative abundance (%) of the phylum (A) and family (B) taxons identified in the cow rumen sample and enriched rumen anaerobic consortium (ERAC). Abundances were determined based on the 16S rRNA gene amplicon sequences. Phyla represented by less than 1% and families represented by less than 3% of the total reads were combined in the groups named “Phyla < 1%” and “Families < 3%,” respectively.

Figure 3 shows the results of the taxonomic analyses of the cow rumen and ERAC based on representative OTU sequences. In the cow rumen, 16 phyla, 22 classes, 28 orders, 42 families, and 69 genera were detected (Fig. 3; Data Set S1). At the phylum level, *Bacteroidetes* and *Firmicutes* were the dominant phyla, comprising 46.1% and 45.0% of the total sequences, respectively. Following this trend, *Bacteroidales* and *Clostridiales* were the most dominant orders, with *Prevotellaceae* and *Clostridia* representing the prevalent families.

The taxonomic profile and the relative abundance of the phylogenetic groups of the ERAC were significantly different from those of the original microbial community (cow rumen sample), a result consistent with the richness and diversity described above. By comparing the taxonomic profile of ERAC to that of the original ruminal sample, the impact of microbial enrichment was detected, whereby the number of phyla decreased from 16 to 9, and there was a considerable enrichment of *Firmicutes* and *Synergistetes* (Fig. 3A; Data Set S1). In comparison to the original sample, the total proportion of sequences assigned to the *Firmicutes* increased from 45.0% to 72.6%, whereas that of sequences assigned to the *Synergistetes* increased from 0.1% to 10.4% (Fig. 3A). In contrast, the proportion of sequences related to the *Bacteroidetes* decreased from 46.1% to 11.6% (Fig. 3A). Within the phylum *Synergistetes*, *Dethiosulfovibrionaceae* represented the most enriched family, comprising more than 9% of the community (Fig. 3A). The enrichment also led to a shift in low-rank taxons; for instance, the *Veillonellaceae* and *Clostridiales* were enriched in the ERAC, making up 47.4% and 17.9% of the community, respectively (Fig. 3B). In contrast, the proportion of sequences of the *Lachnospiraceae* decreased from 13.0% to 1.6%. However, the proportion of sequences of the *Prevotellaceae* decreased to 0.3%, whereas the proportion of sequences of the lineage belonging to the *Bacteroidetes* increased to 9.3% of the community (Fig. 3B).

### Metagenome sequencing and assembly.

Metagenome shotgun sequencing of the ERAC yielded 21 million high-quality paired-end reads, representing 3.1 GB of sequences. Using *de novo* assembly, 88.2% of the reads were assembled into 103,541 contigs varying in size from 200 to 978,274 bp (*N*_50_, 21,714). The gene prediction depicted 142,703 protein-coding sequences. To gain insight into the diverse biochemistry potential of the ERAC, a gene-centric metagenome analysis was carried out based on the Clusters of Orthologous Groups (COG), KEGG, and Pfam annotations.

A total of 99,763 (69.9%) predicted genes were classified according to COG categories, 63,855 (44.7%) were identified in the KEGG database, and 95,457 (66.9%) had at least one protein domain predicted according to the Pfam database. Although the annotation based on COG identified more genes than the KEGG analysis, both sets of results indicated that most of the protein-coding genes were classified in the metabolism category (Fig. S3 and S4). Within the metabolism category, a high proportion of genes was associated with carbohydrate and amino acid metabolism.

Additionally, we applied a Pfam-based analysis, as described previously (26), to investigate whether conserved domains related to lignin and aromatic degradation were present in the ERAC metagenome data. Domains of peroxidases, laccases, catalases, as well enzymes that cleave lignin linkages, such as β-aryl ether bonds, biphenyl linkages, and hydroxyl groups (*ortho* cleavage), were found in the ERAC metagenomic data (Table S4), suggesting the potential for lignin degradation.

### CAZyme profile of the ERAC.

To investigate the anaerobic consortium genomic content for plant biomass breakdown, the ERAC metagenome sequences were screened against the hidden Markov model (HMM) profile-based database dbCAN (32). According to the CAZy database classification scheme, of the 142,703 predicted proteins, 5,070, representing 3.5% of the total predicted proteins, were predicted to have at least one carbohydrate-active function. The ERAC metagenome contains 2,158 glycoside hydrolase (GHs) modules, 695 carbohydrate-binding modules (CBMs), 17 cohesin modules, 159 dockerin modules, 1,457 glycosyltransferase (GT) modules, 858 carbohydrate esterase (CE) modules, 69 polysaccharide lyase (PL) modules, 176 auxiliary activity (AA) modules, and 175 S-layer homology (SLH) modules. An overview of all predicted families in the CAZy database is described in Table 1, as well as in Data Set S2 in the supplemental material.

**TABLE 1:** The most common CAZyme modules predicted in the total ERAC metagenome and their relative abundance in ERACgs, according to their representation in the CAZy database^*a*^

Family	No. of CAZyme modules	
	Total metagenome	ERACgs
Most common GH families		
GH13	181	147
GH3	126	100
GH2	117	101
GH43	117	84
GH23	84	56
GH5	69	47
GH25	67	47
GH77	56	41
GH31	52	39
Most common CBM families		
CBM50	187	139
CBM32	98	80
CBM48	66	53
CBM6	33	15
CBM67	29	27
Most common CE families		
CE1	223	139
CE10	173	122
CE4	148	110
CE3	92	60
CE9	43	28
CE1	223	139
Most common PL families		
PL12	18	13
PL22	14	14
PL1	11	7
Most common AA families		
AA6	136	83
AA3	17	10

a Abbreviations: ERAC, enriched rumen anaerobic consortium; ERACgs, enriched rumen anaerobic consortium genomes; CAZyme and CAZy, carbohydrate-active enzyme; GH, glycoside hydrolase; CBM, carbohydrate-binding module; CE, carbohydrate esterases; PL, polysaccharide lyases; AA, auxiliary activities.

Analyzing in more detail the CAZyme prediction, the ERAC contained 92 distinct GH families. Among them, we found GH families encoding cellulases, oligosaccharide-degrading enzymes, mannases, pectinases, chitinases, α-amylases, and xylanases. The remaining CAZyme families identified in the ERAC, such as CE, PL, and AA families (Data Set S2), also play important roles in lignocellulose breakdown (33, 34). Among them, we found families encoding enzymes for xylan, pectin, and alginate degradation. Furthermore, we noticed nonhydrolytic accessory CBMs, which are protein domains found in carbohydrate-active enzymes that can potentiate the activity of the associated catalytic domains (33). The set of predicted CBMs in the ERAC comprised 43 families, including CBMs that bind to xylan, cellulose, starch, pullulan, and glucans (Table 1 and Data Set S2).

Overall, the ERAC is composed of microorganisms carrying a wide variety of carbohydrate-degrading genes with the potential to produce a broad range of enzymatic activities to deconstruct all components of the plant cell wall. A complete description of the families and their corresponding enzymatic activities is given in the supplemental material.

### Novel CAZymes and prediction of multimodular proteins.

**FIG 4:**
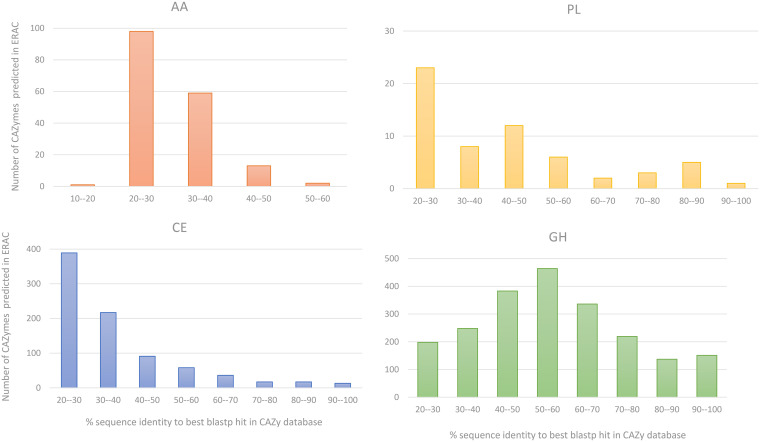
Distribution of the percent identity of the carbohydrate-active enzyme (CAZyme) sequences predicted in the enriched rumen anaerobic consortium (ERAC) against the four classes of the CAZy database. Only the maximum percent identities for each CAZyme ERAC were considered. GH, glycoside hydrolase; PL, polysaccharide lyases; CE, carbohydrate esterases; AA, auxiliary activities.

To confirm the novelty of the enzymes identified in this study, the CAZyme content in the ERAC was compared to the entries in the CAZy database (as described previously [23]). Considering the GH, CE, PL, and AA classes, which are classes more often involved in biomass breakdown, we found that 3,042 CAZyme sequences predicted in the ERAC (60% of the total) had less than 90% identity to the amino acid sequences reported in the CAZy database (Fig. 4). These CAZyme sequences include cellulases, xylanases, pectate lyases, carbohydrate esterases, etc. Interestingly, among the CAZyme classes depicted in the ERAC, the AA family members had the lowest similarity match compared to that of the other families in the CAZy database (Fig. 4).

CAZymes tend to be modular proteins composed of both catalytic and noncatalytic accessory domains (e.g., CBMs, dockerin modules, or SLH modules) (35). The presence of noncatalytic domains appended to CAZymes indicates (i) improved enzymatic efficiency due to a substrate proximity effect mediated by the binding domain or (ii) that CAZymes may be organized in enzymatic complexes or free-enzyme systems. We further investigated whether the CBM, dockerin, and SLH sequences from ERAC were appended to catalytic CAZyme domains, forming multiple-domain proteins. Approximately 14% (711) of the GH, CE, and PL sequences in the ERAC were predicted to have at least one additional domain, indicating that the ERAC CAZymes may be organized in enzymatic complexes or free-enzyme systems (Tables S5 to S7 and Data Set S2).

Of the predicted CBM sequences, 53% of the sequences were appended to CAZymes, forming 165 distinct types of genetic multimodular structures (Tables S1 and S5). Thirty-seven GH, 7 CE, and 2 PL family members contained dockerin modules; in addition, CBM families were depicted in these protein sequences. The multimodular CAZymes identified in the ERAC were also previously related to the degradation of starch (CBM48-GH13_9 and CBM34-GH13_2), pectinases (CBM67-GH78), acetylated polysaccharides (CBM48-CE1), and oligosaccharides (GH43_35-CBM6) (36–38). The most frequent multidomain protein sequences found in the ERAC were CBM48-GH13_9, CBM67-GH78, CBM34-GH13_20, and CBM48-CE1.

Of the multimodular dockerin-containing proteins with a predicted catalytic function, the most prevalent sequences were found to be appended to peptidase domains. Previous studies suggest that this modular organization may be involved in microbial competition or may permit these enzymes to act in synergy with cellulases for carbohydrate processing (39, 40). Several dockerin modules were also predicted to be appended to the CE and/or GH families in the ERAC, indicating that these sequences are linked to potential cellulosomes. In addition, several sequences harboring cohesin and SLH modules were identified, providing additional evidence of microorganisms within the ERAC able to produce cellulosomes.

The remaining CBM, dockerin, or SLH sequences appended to domains without a predicted function were further subjected to Pfam domain annotation using the WebMGA web server (41) to classify domains of unknown function (DUF). The analysis of multimodular proteins comprising DUF appended to noncatalytic accessory domains is a relevant approach for the discovery and exploitation of new CAZyme family members (23). From the DUF screening strategy, we identified 28 DUFs appended to nine CBM family, dockerin module, and SLH module sequences, comprising 30 different types of domain organizations (Table S7).

### Reconstructed genomes with a potential lignocellulolytic capacity.

In addition to metagenome assembly, the reconstruction of genomes directly from metagenome data sets has become a powerful strategy to link the metabolic and functional potential with phylogenetic information (8). The metagenome-assembled genomes (MAGs), named enriched rumen anaerobic consortium genomes (ERACgs), were assessed in terms of their completeness and contamination, based on the presence or absence of sets of colocalized single-copy marker genes within a reference genome tree (42). This resulted in 19 ERACgs that were nearly complete (≥90% completeness), 19 that were substantially complete (≥70%), and 3 that were moderately complete (≥50%) (Table 2). Based on the same criteria, 4 ERACgs that displayed a low contamination level (≤2%) were maintained in the subsequent analysis. The size of the ERACgs ranged from 1.39 and 4.51 MB, the GC content varied from 28.3 to 66.5%, and between 1,245 and 3,935 coding sequences (CDS) were predicted (Table 2).

**TABLE 2:** Genomic features of ERACgs from ERAC metagenome shotgun sequencing^*a*^

ERACg identifier	Phyla-AMPHORA classification		Completeness (%)	Contamination (%)	Genome size (Mb)	Predicted no. of genes	GC content (%)	No. of CAZymes^*b*^
	Class	Predicted taxon						
ERACg_2	*Clostridia*	*Butyrivibrio*	96.6	0	2.65	2,421	38.2	106
ERACg_3	*Clostridia*	*Ruminiclostridium*	95.3	0	2.76	2,608	49.4	63
ERACg_5	*Clostridia*	*Clostridium*	89.2	0	2.29	1,970	51.2	50
ERACg_9	*Clostridia*	*Clostridium*	92.6	0	3.53	3,317	57.5	186
ERACg_11	*Clostridia*	*Butyrivibrio*	95.9	0	2.99	2,705	43.6	156
ERACg_12	*Clostridia*	*Oscillibacter*	91.9	0	2.36	2,169	52.5	52
ERACg_13	*Clostridia*	*Oscillibacter*	78.4	0	2.42	2,356	62.9	81
ERACg_15	*Clostridia*	*Clostridiales*	83.8	0	2.30	2,277	55	50
ERACg_16	*Clostridia*	*Oscillibacter*	92.6	0	1.97	1,904	59.5	42
ERACg_21	*Clostridia*	*Clostridium*	84.5	0	2.78	2,576	56.4	99
ERACg_23	*Clostridia*	*Clostridium*	81.7	2.5	3.56	3,485	31	101
ERACg_25	*Clostridia*	*Desulfitobacterium*	95.3	0	2.57	2,387	39.1	49
ERACg_26	*Clostridia*	*Oscillibacter*	93.2	0	2.35	2,275	60.2	61
ERACg_32	*Clostridia*	*Butyrivibrio*	93.9	0	3.09	2,790	45.2	168
ERACg_42	*Clostridia*	*Ruminococcus*	95.3	0	2.79	2,496	48.3	180
ERACg_45	*Clostridia*	*Alkaliphilus*	91.2	0	2.31	2,292	30.5	65
ERACg_48	*Clostridia*	*Filifactor*	88.5	0	1.75	1,665	47.6	55
ERACg_50	*Clostridia*	*Clostridium*	91.9	0	2.59	2,624	28.3	50
ERACg_57	*Clostridia*	*Butyrivibrio*	93.9	0	4.48	3,824	42.4	135
ERACg_58	*Clostridia*	*Clostridiales*	91.2	0	1.57	1,409	56	12
ERACg_41	*Bacilli*	*Streptococcus*	97.3	0	2.05	1,823	51.2	56
ERACg_8	*Bacilli*	*Enterococcus*	96.6	0	3.11	2,772	53.9	50
ERACg_1	*Erysipelotrichia*	*Erysipelothrix*	87.8	0	1.39	1,245	32.2	43
ERACg_19	*Bacteroidia*	*Prevotella*	62.8	0	1.81	1,468	52.7	115
ERACg_30	*Bacteroidia*	*Porphyromonadaceae*	83.1	0	2.25	1,861	49.1	164
ERACg_35	*Bacteroidia*	*Bacteroides*	91.9	0	2.23	1,924	50	113
ERACg_37	*Bacteroidia*	*Bacteroides*	84.5	0	3.14	2,574	46	136
ERACg_43	*Bacteroidia*	*Bacteroides*	92.6	0	3.95	3,136	46.6	336
ERACg_55	*Bacteroidia*	*Prevotella*	79.1	0	2.54	2,081	56	128
ERACg_56	*Bacteroidia*	*Prevotella*	68.2	0	2.05	1,650	53.6	140
ERACg_14	*Spirochaetia*	*Sphaerochaeta*	85.8	0	2.63	2,307	54.9	78
ERACg_31	*Spirochaetia*	*Treponema*	81.8	0.7	3.13	2,774	36.5	112
ERACg_36	*Spirochaetia*	*Sphaerochaeta*	81.8	1.7	2.59	2,431	50	82
ERACg_52	*Spirochaetia*	*Treponema*	80.4	0	2.76	2,364	38.3	81
ERACg_4	*Synergistia*	*Aminobacterium*	65.5	0	4.51	3,901	43.3	358
ERACg_38	*Synergistia*	*Aminobacterium*	89.9	0	4.07	3,935	44.5	72
ERACg_49	*Synergistia*	*Aminobacterium*	91.2	0	2.24	2,154	41.5	47
ERACg_18	*Deltaproteobacteria*	*Proteobacteria*	76.4	1.22	2.21	2,175	57.4	41
ERACg_34	*Deltaproteobacteria*	*Desulfovibrio*	85.8	0	2.69	2,260	64.7	61
ERACg_54	*Deltaproteobacteria*	*Desulfovibrio*	89.2	0	3.35	3,152	66.5	99
ERAC_46	*Alphaproteobacteria*	*Rhizobium*	85.8	0	2.49	2,428	60.8	43

a Abbreviations: ERACg, enriched rumen anaerobic consortium genomes; ERAC, enriched rumen anaerobic consortium.

b Total number of carbohydrate-active enzymes (CAZymes) predicted.

The ERACgs were assigned to the lowest taxonomic level that could be confidently determined by phylogenetic marker genes. The phylum *Firmicutes*, the predominant phylogenetic group, was represented by 20 ERACgs assigned to the *Clostridia* class, followed by the *Bacilli* (2 ERACgs) and *Erysipelotrichia* (1 ERACg) classes. The second and third most abundant groups were assigned to the *Bacteroidia* (7 ERACgs) and *Spirochaetia* (4 ERACgs) classes. The remaining ERACgs were assigned to the *Synergistia* (3 ERACgs), *Deltaproteobacteria* (3 ERACgs), and *Alphaproteobacteria* (1 ERACg) classes.

**FIG 5:**
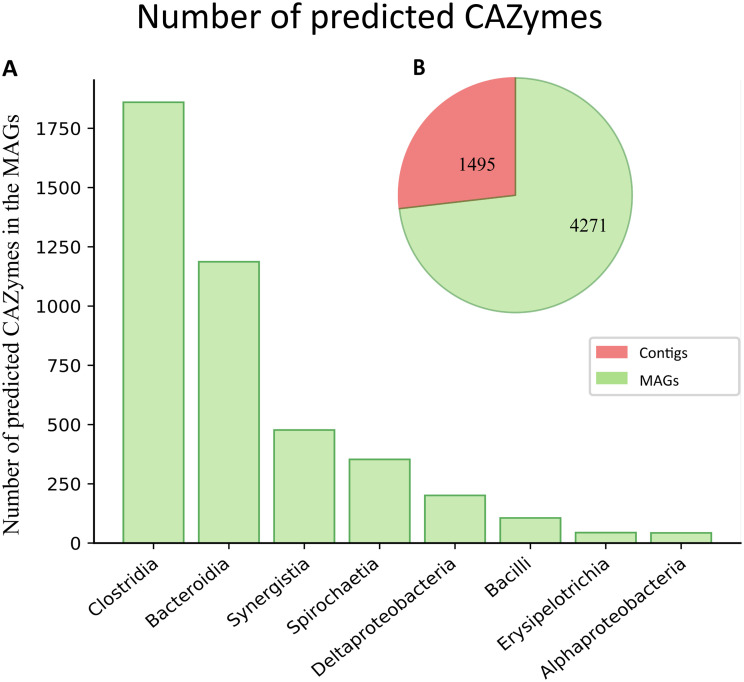
(A) Distribution of the predicted carbohydrate-active enzymes (CAZymes) found in enriched rumen anaerobic consortium genomes (ERACgs) at the class level. (B) Total CAZymes found in the rumen-derived anaerobic microbial consortium (enriched rumen anaerobic consortium [ERAC]) metagenome data. Red, nonbinned metagenome contigs; green, ERACgs.

The ERACg genetic content related to lignocellulose hydrolysis was investigated in detail. The ERACgs contained approximately 72% of the total predicted CAZymes in the ERAC (Fig. 5). *Clostridia* and *Bacteroidia* ERACgs harbored the highest number of predicted GHs (Fig. 5; Data Set S2), accounting for 56% (1,207 out of 2,158) of the total number of GH domains encountered in the ERAC. Seven (out of 20) *Clostridia* ERACgs and all *Bacteroidia* ERACgs harbored more than 100 CAZymes (Table 2). These two phylogenetic groups encoded 65 and 66 distinct GH families (Data Set S2), respectively.

**FIG 6:**
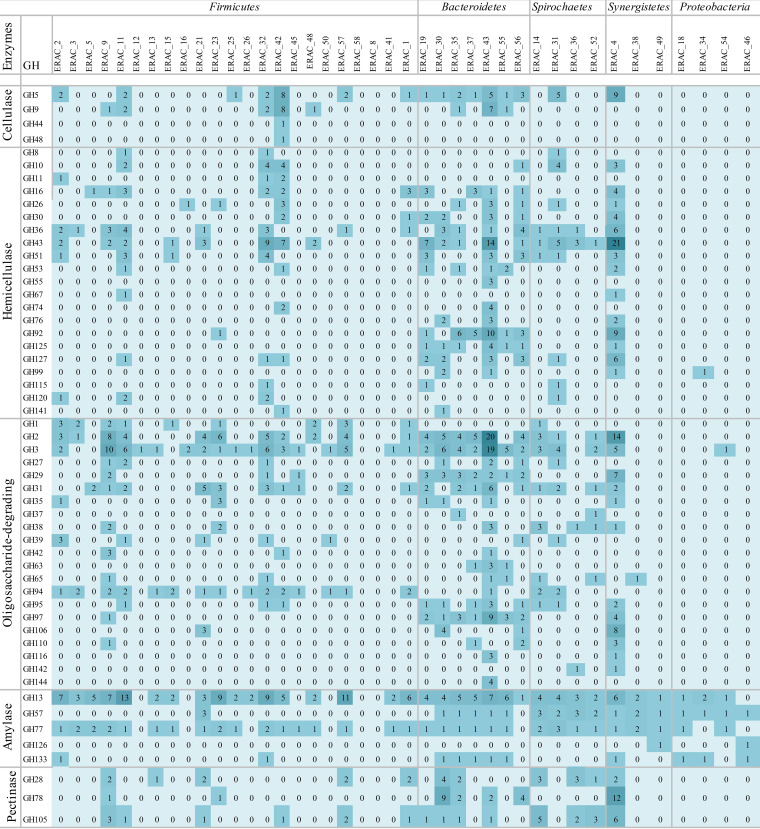
Heat map displaying the distribution of the most abundant glycoside hydrolases (GH^1^) found in the ERACgs from ERAC. GH families were grouped according to their action on components of the plant cell wall.

In general, *Clostridia* and *Bacteroidia* ERACgs harbored a diverse repertoire of GHs which was capable of degrading cellulose, hemicellulose, starch, and pectin (Fig. 6). Although the cellulases were not among the most abundant GH domains in the ERACgs, six distinct families were depicted: GH5, GH9, GH30, GH51, GH74, and GH94. These were predicted mainly in *Clostridia* and *Bacteroidia* ERACgs (Fig. 6; Data Set S2). These ERACgs also showed the highest abundance of the CE, PL, and AA families (Data Set S2).

The high diversity of CAZyme families was also observed in the remaining ERACgs (*Spirochaetia*, *Synergistia*, and *Proteobacteria*). The *Spirochaetia* and *Synergistia* ERACgs possessed 39 and 49 distinct GH families, respectively (Fig. 6; Data Set S2). Nonetheless, the numbers of CAZymes predicted in these groups were not high. These groups accounted for 19.4% of the total GH count predicted in the ERAC. Within this group, only *Treponema* sp. ERACg_31 and *Aminobacterium* sp. ERACg_4 encoded more than 100 CAZymes (Table 2). Moreover, *Aminobacterium* sp. ERACg_4 harbored the highest number of predicted CAZymes among ERACgs, encoding 358 CAZymes, indicating a full capacity to fully degrade plant cell wall polysaccharides.

By comparing the enzymatic sets among the phylogenetic groups, in general, *Spirochaetia* ERACgs had a potential capacity to degrade biomass similar to that of *Clostridia* and *Bacteroidia* ERACgs (Fig. 6). The remaining *Synergistia* and *Proteobacteria* ERACgs had an enzymatic set restricted to the degradation of starch.

### Macromolecular enzymatic complexes: cellulosomes and PULs.

Besides the CAZyme profile, we also investigated the ability of the ERACgs to produce multienzyme complexes, such as cellulosomes and PULs. These multidomain macromolecular enzymatic complexes are highly efficient metabolic systems that break down polysaccharide complex substrates (16, 43–45). The cellulosomes comprise a combination of dockerin-bearing catalytic domains that bind the cohesin modules, which are part of noncatalytic structural proteins called scaffoldins (14). Moreover, cellulosomes can be subdivided into both simple and highly structured multidomain macromolecule structures, which are composed of more than one scaffoldin protein (45). The PULs comprise a series of linked genes encoding all activities necessary to bind, transport, and depolymerize a broad type of glucan (16). The PULs are organized around tandem *susCD*-like pairs encoding integral membrane proteins and extracellular lipoproteins.

**FIG 7:**
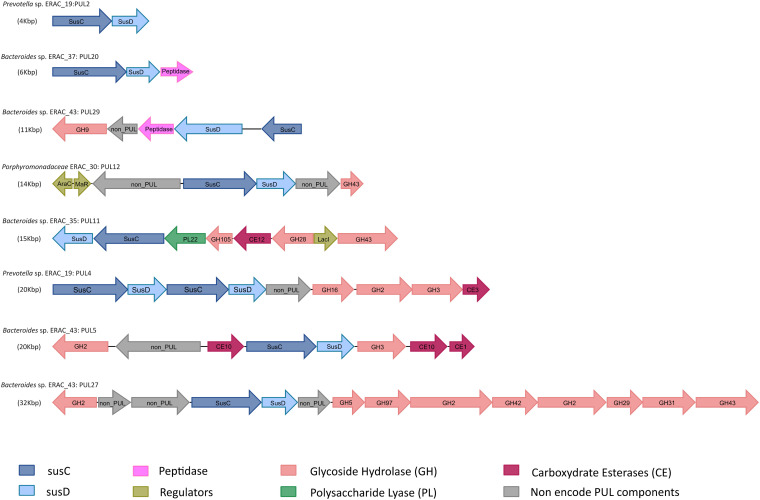
Examples of polysaccharide utilization loci (PUL) predicted in *Bacteroidia* ERACgs reconstructed from the enriched rumen anaerobic consortium metagenome. To facilitate the visualization of gene arrangements, the predicted proteins were colored according to the function of the encoded proteins: SusC, SusD, glycoside hydrolase (GH), polysaccharide lyase (PL), carbohydrate esterase (CE), peptidase, and regulators (AraC, MaR, LacI). Genes that do not encode PUL components or that encode hypothetical proteins are identified as non-PUL genes. All PULs predicted in *Bacteroidia* ERACgs are presented in Data Set S2 in the supplemental material.

Potential cellulosomes and PULs were both identified among the ERACg genes encoding cellulosomal proteins (cohesin and dockerin modules) and SusCD-like pairs, respectively. In addition, these protein sequences were manually curated based on BLASTp analysis to confirm the identity of the conserved protein domains. The screen revealed four ERACgs (ERACg_32, ERACg_42, ERACg_50, and ERACg_57) assigned to *Clostridia* encoding putative scaffoldins (Table S8) and all *Bacteroidia* ERACgs encoding PULs (Fig. 7; Data Set S3). Among the potential cellulosome-producing *Clostridia* ERACgs, a detailed analysis indicated that the only *Ruminococcus* sp. ERACg_42 has multimodular CAZymes that co-occur with dockerin, which is essential for the assembly of the cellulosomes (45), thus representing a unique ERACg able to produce cellulosomes.

Regarding PUL prediction, a total of 154 PULs were identified in all ERACgs assigned to *Bacteroidia*, and the number per genome varied from 3 to 50 (Fig. 7; Data Set S3). ERACg_37 and ERACg_43, both assigned to the *Bacteroides* genus, contained 39 and 50 PULs, respectively, representing the ERACgs with the highest number of predicted PULs. The remaining ERACgs harbored fewer PULs, such as *Prevotella* sp. ERACg_19 (10 PULs), *Porphyromonadaceae* ERACg_30 (27 PULs), *Bacteroides* sp. ERACg_35 (12 PULs), *Prevotella* sp. ERACg_55 (13 PULs), and *Prevotella* sp. ERACg_56 (3 PULs). Sixty-nine PULs were associated with genes encoding CAZymes, peptidases, transporters, and transcriptional regulators (e.g., hybrid two-component systems [HTCS], AraC, GntR), indicating the presence of complete systems capable of degrading polysaccharide and proteins (Data Set S3). We counted 47 distinct CAZyme families associated with PULs, implying that PULs may be able to degrade many kinds of complex lignocellulose substrates. Among the CAZyme predictions associated with PULs, we encountered putative cellulases (GH5 and GH9), amylases (GH13 and GH97), mixed-linkage β-glucanases (GH16), and oligosaccharide-degrading enzymes (GH3 and GH31) (Data Set S3). The CE families, such as CE1, CE6, CE10, and CE12, were also associated with a tandem *susCD* gene pair.

An illustrative example of the PUL diversity found in the different ERACgs is shown in Fig. 7. Some PULs are composed of enzymes targeting specific substrates or a broader pool of substrates. For example, ERACg_46 harbors a cluster (PUL27) encoding seven different CAZymes, of which five are oligosaccharide-degrading enzymes (GH2, GH29, GH31, GH42, and GH97), one is cellulase (GH5), and the last one is potentially involved in xylan degradation (GH43), whereas PUL4 from *Prevotella* sp. ERACg_19 encodes enzymes that degrade hemicellulose (GH16 and CE3) and oligosaccharides (GH2 and GH3). *Prevotella* sp. ERACg_19 also has other clusters (PUL29) composed of genes encoding enzymes for cellulose (GH9) and protein degradation (peptidase).

Previously, metagenome analysis of cow rumen (46) and moose rumen (6) found PULs containing dockerin modules appended to GHs. In our study, we also found dockerin-containing proteins in *Prevotella* sp. ERACg_55 and *Prevotella* sp. ERACg_56, which were ERACgs affiliated with the *Bacteroidia* class. These dockerin-containing genetic structures were appended to GH modules, DUFs, and CBM modules, but none were found to be associated with PULs. Although the presence of dockerin modules in PULs from rumen *Bacteroidetes* was previously reported (6, 46), the functional role of these modules in this genetic context is not defined yet.

### Metaproteome for ERAC.

Metaproteome analysis is a powerful strategy to illustrate which phylotypes are actively producing enzymes in microbial communities. The approach proposes a direct link between biotechnologically relevant enzyme activity and the corresponding gene encoding the enzyme (22). To experimentally reveal the set of CAZymes found from the consortium metaproteome, as well as to confirm the production of cellulosomes, we applied a mass spectrometry-based method. For this purpose, the culture supernatant was taken for metaproteome analysis after 5 days of growth in fresh medium (after 25 cycles of medium transfer).

A total of 334 proteins were detected in the ERAC metaproteome (Data Set S4). Analysis of the taxonomic origin of the secreted proteins confirmed that 36 of the ERACgs identified in the ERAC metagenomic data were metabolically active. Nonetheless, examining in detail the function and distribution of the secreted proteins, *Ruminococcus* sp. ERACg_42 in the consortium showed the highest number of different proteins identified in the metaproteome, representing 39.5% of the total proteins detected (Tables 3 and 4; Tables S9 and S10). Most proteins secreted by *Ruminococcus* sp. ERACg_42 were related to cellulosomal proteins, indicating the production of cellulosomes.

**TABLE 3:** Putative cellulosomal proteins and SusC/SusD families identified by LC-MS/MS from ERAC grown on sugarcane bagasse^*a*^

ERACg identifier	Predicted protein	Modular architecture	Signal peptide^*b*^	Total spectral count^*c*^
*Butyrivibrio* sp. ERACg_32	Cellulosomal protein	CBM6-CBM6-CBM6-CBM6-CBM2	Yes	14
*Ruminococcus* sp. ERACg_42	Putative scaffoldin	6× cohesin_I-CttA	Yes	10
*Ruminococcus* sp. ERACg_42	Putative scaffoldin	Cohesin	Yes	23
*Ruminococcus* sp. ERACg_42	Putative scaffoldin	Cohesin_III	Yes	5
*Ruminococcus* sp. ERACg_42	Putative scaffoldin	Cohesin_I-dockerin_I	Yes	19
*Ruminococcus* sp. ERACg_42	Putative scaffoldin	Dockerin_I-Cthe_2159-Cthe_2159	Yes	14
*Ruminococcus* sp. ERACg_42	Putative scaffoldin	Dockerin_III-cohesin_III-Dockerin_I	Yes	5
*Ruminococcus* sp. ERACg_42	Putative scaffoldin C	No domain	Yes	2
*Ruminococcus* sp. ERACg_42	Putative scaffoldin	No domain	Yes	49
*Ruminococcus* sp. ERACg_42	Cellulosomal protein	Dockerin_I	Yes	4
*Ruminococcus* sp. ERACg_42	Cellulosomal protein	LRR_5-dockerin_I	Yes	19
*Ruminococcus* sp. ERACg_42	Cellulosomal protein	LRR_5-dockerin_I	Yes	1
*Ruminococcus* sp. ERACg_42	Cellulosomal protein	LRR_5-dockerin_I	Yes	6
*Ruminococcus* sp. ERACg_42	Cellulosomal protein	LRR_5-dockerin_I	Yes	2
*Ruminococcus* sp. ERACg_42	Cellulosomal protein	DUF4874-DUF4832-dockerin_I	Yes	1
*Ruminococcus* sp. ERACg_42	Peptidase	Dockerin_I-peptidase	Yes	1
*Ruminococcus* sp. ERACg_42	Cellulosomal protein	Dockerin_I	Yes	6
*Bacteroides* sp. ERACg_43	SusD family protein		No	1
*Bacteroides* sp. ERACg_43	Starch binding associated with outer membrane		No	1
*Bacteroides* sp. ERACg_43	TonB-linked outer membrane protein, SusC/RagA family		No	1
*Bacteroides* sp. ERACg_43	TonB-linked outer membrane protein, SusC/RagA family		No	1
*Bacteroides* sp. ERACg_43	TonB-linked outer membrane protein, SusC/RagA family		No	6
*Bacteroides* sp. ERACg_43	TonB-linked outer membrane protein, SusC/RagA family		No	1
*Bacteroides* sp. ERACg_43	SusD family protein		No	1
*Bacteroides* sp. ERACg_43	TonB-linked outer membrane protein, SusC/RagA family		No	6

a Abbreviations: cohesin_number, cohesin type number; dockerin_number, dockerin type number; Cthe_2159 represents a novel family of cellulose-binding beta-helix proteins from *Clostridium thermocellum*; LRR_5, leucine-rich repeats; PUL, polysaccharide utilization loci. Cohesin and dockerin domains are represented with the family number according to their representation in the dbCAN database. The protein set secreted by enriched rumen anaerobic consortium (ERAC) is given in Data Set S3 in the supplemental material.

b Prediction of signal peptides based on SignalP analysis.

c Metaproteome analysis based on spectral counting.

**TABLE 4:** CAZy families identified by LC-MS/MS from ERAC grown on sugarcane bagasse^*a*^

ERACg identifier	Predicted protein	Modular architecture	EC no.	Secretion signal^*b*^	Total spectrum count^*c*^
*Ruminococcus* sp. ERACg_42	Endoglucanase	CBM79-CBM79-GH5_4	3.2.1.4	Yes	8
*Ruminococcus* sp. ERACg_42	Endoglucanase	GH5_1-dockerin_I	3.2.1.4	Yes	7
*Ruminococcus* sp. ERACg_42	Endoglucanase	GH5_1-dockerin_I	3.2.1.4	Yes	17
*Ruminococcus* sp. ERACg_42	Cellulase	GH9-CBM3-dockerin_I	3.2.1.4	Yes	14
*Ruminococcus* sp. ERACg_42	Cellulase:acetylxylan esterase	GH5_4-CBM22-CE3-dockerin_I	3.2.1.4, 3.1.1.72	Yes	7
*Ruminococcus* sp. ERACg_42	Cellulase	CBM4-CBM30-GH9-dockerin_I	3.2.1.4	Yes	2
*Ruminococcus* sp. ERACg_42	Cellulase	GH9-CBM3-dockerin_I	3.2.1.4	Yes	8
*Ruminococcus* sp. ERACg_42	Endoglucanase	GH9-CBM79-dockerin_I	3.2.1.4	Yes	39
*Ruminococcus* sp. ERACg_42	Cellulase	GH5_4-CBM80-dockerin_I	3.2.1.4	Yes	69
*Ruminococcus* sp. ERACg_42	Cellulase	GH9-CBM3-dockerin_I	3.2.1.4	Yes	8
*Ruminococcus* sp. ERACg_42	Cellulase	GH9-CBM3-dockerin_I	3.2.1.4	Yes	32
*Ruminococcus* sp. ERACg_42	Glycoside hydrolase family 44	GH44-CBM76-dockerin_I	Not determined	Yes	2
*Ruminococcus* sp. ERACg_42	Cellulase:acetylxylan esterase	GH5_4-CBM22-CE3-Dockerin_I	3.2.1.4, 3.1.1.72	Yes	7
*Ruminococcus* sp. ERACg_42	Mannan endo-1,4-β-mannosidase	CBM35-GH26-dockerin_I	3.2.1.78	Yes	1
*Ruminococcus* sp. ERACg_42	Xyloglucan-specific endo-β-1,4-glucanase	GH5_4-CBM22-dockerin_I	3.2.1.151	Yes	14
*Ruminococcus* sp. ERACg_42	Glucuronoarabinoxylan endo-1,4-β-xylanase; feruloyl esterase	GH5_4-CBM22-dockerin_I	3.2.1.136, 3.1.1.73	Yes	11
*Ruminococcus* sp. ERACg_42	Endo-1,4-β-xylanase; feruloyl esterase	GH10-CBM22-CE1	3.2.1.8, 3.1.1.73	Yes	11
*Ruminococcus* sp. ERACg_42	Endo-1,4-β-xylanase; nonreducing end α-l-arabinofuranosidase	CBM22-GH10-CBM22-dockerin_I-GH43-CBM36	3.2.1.8, 3.2.1.55	Yes	37
*Ruminococcus* sp. ERACg_42	Endo-1,4-β-xylanase	CBM22-GH10-dockerin_I	3.2.1.8	Yes	3
*Ruminococcus* sp. ERACg_42	Endo-1,4-β-xylanase; feruloyl esterase	GH43_10-CBM22-dockerin_I-CE1	3.2.1.37, 3.1.1.73	Yes	15
*Ruminococcus* sp. ERACg_42	Xylan-1,4-β-xylosidase	GH43_29-CBM6-CBM22-dockerin_I	3.2.1.37	Yes	2
*Ruminococcus* sp. ERACg_42	Oligoxyloglucan reducing-end-specific cellobiohydrolase	GH74-dockerin_I	3.2.1.150	Yes	12
*Ruminococcus* sp. ERACg_42	Endo-β-1,4-xylanase; chitin deacetylase	GH11-CBM22-dockerin_I-CBM22-CE4	3.2.1.8, 3.5.1.41	Yes	1
*Ruminococcus* sp. ERACg_42	Arabinan endo-1,5-α-l-arabinosidase	GH43-CBM13-dockerin_I	3.2.1.99	Yes	12
*Ruminococcus* sp. ERACg_42	Mannan endo-1,4-β-mannosidase	CBM35-GH26-dockerin_I	3.2.1.78	Yes	1
*Ruminococcus* sp. ERACg_42	Acetylxylan esterase	Dockerin_I-CE2-CBM4	3.1.1.72	Yes	1
*Ruminococcus* sp. ERACg_42	Putative glycoside hydrolase family 141	GH141-CBM6-dockerin_I	Not determined	Yes	1
*Ruminococcus* sp. ERACg_42	β-Galactosidase	GH2-dockerin_I	3.2.1.23	Yes	8
*Ruminococcus* sp. ERACg_42	Carbohydrate esterase family 12	CE12-CBM13-dockerin_I-CBM35-CE12	3.1.1.86	Yes	2
*Ruminococcus* sp. ERACg_42	Rhamnogalacturonan endolyase	PL11-dockerin_I	4.2.2.23	Yes	13
*Ruminococcus* sp. ERACg_42	Pectate lyase	PL1-PL9-dockerin_I	4.2.2.2; 4.2.2.9	Yes	3
*Aminobacterium* sp. ERACg_4	Glycoside hydrolase family 18	GH18	Not determined	Yes	1

a Abbreviations: ERAC, enriched rumen anaerobic consortium; ERACg, enriched rumen anaerobic consortium genome; EC, Enzyme Commission; cohesin_number, cohesin type number; dockerin_number, dockerin type number; GH, glycoside hydrolase; CBM, carbohydrate-binding module; CE, carbohydrate esterases; PL, polysaccharide lyases; ND, not determined. CAZymes are represented with the family number according their representation in the CAZy database.

b Prediction of signal peptides based on SignalP analysis.

c Metaproteome analysis based on spectral counting.

Besides the identification of the cellulosomes, the metaproteome analysis also experimentally confirmed a second enzymatic complex, a PUL from *Bacteroides* sp. ERACg_43, which was also predicted from the ERAC metagenome data (Table 3). Although the CAZymes were not detected from *Bacteroides* sp. ERACg_43 in this analysis, the identification of SusCD proteins proves that this enzymatic complex is produced by this phylotype.

### Taxonomic and CAZyme analyses of *Ruminococcus* ERACg_42.

*Ruminococcus* species, which fall within the phylum *Firmicutes*, are found in anaerobic environments, including the human gut (e.g., Ruminococcus champanellensis [47]), biogas (e.g., Clostridium bornimense [48]), and rumen (e.g., R. flavefaciens [49]). Some *Ruminococcus* isolates are described to be cellulosome-producing bacteria, thus representing important microorganisms for biotechnological application related to biofuel production from lignocellulosic biomass (49–51).

Based on our taxonomic classification, ERACg_42 belongs to the *Ruminococcus* genus. The classification was carried out based on two different methods, the use of marker genes (the Phyla-AMHORA classification [48]) and alignment of *k*-mers (Kraken classification [49]). Nonetheless, an additional phylogenomic analysis was performed to avoid unequivocal taxonomic classification and to reveal genomic features common to the *Ruminococcus* genus. This analysis is based on orthologous genes among the genomes of different species indicating rearrangements, deletions, and insertions in the chromosomes and determining the speciation process and its functional consequences (52). Using draft genomes of type strains of the genus *Ruminococcus*, a phylogenetic tree was reconstructed based on 304 concatenated orthologous proteins, illustrating the evolutionary distances among *Ruminococcus* species (Fig. S5). *Ruminococcus* ERACg_42 is closely related to R. flavefaciens ATCC 19208. Both genomes share 1,698 orthologous genes, representing 66.6% and 53.9% of all proteins predicted for *Ruminococcus* ERACg_42 and R. flavefaciens ATCC 19208, respectively (Data Set S5). The coding sequences for cellular processes (e.g., extracellular structures, transporters, cell division) and nucleotide and carbohydrate metabolism are within the core set of genes.

The draft genome of *Ruminococcus* sp. ERACg_42 encodes 72 GHs and at least 11 different loci bearing genes encoding cellulosomal structures. Among the 72 predicted GHs in *Ruminococcus* sp. ERACg_42, 37 of them (50.7%) were from 17 distinct families and harbored type I dockerin modules, and several of them were also found in combination with CBMs (Table S10). These putative cellulosomal genes encode cellulases (GH5, GH9, and GH44), xylanases (GH10, GH11, GH30, and GH127), mannanases (GH26), and arabinogalactan endo-β-1,4-galactanase (GH53). The proteins (37 GHs) encoded by the majority of these putative cellulosomal coding sequences show amino acid identity ranging from 34% to 82% with R. flavefaciens GHs. The proteins encoded by these genes were found to be appended to CBM76, CBM79, and CBM80, which so far have been found exclusively in ruminococcal species (53). We also found CEs and PLs appended to dockerin modules.

The analysis also indicated that *Ruminococcus* sp. ERACg_42 is a producer of cellulosomes. Our analyses depicted 11 scaffoldin protein sequences, 10 of which represented putative scaffoldin proteins harboring type I or III cohesin modules (Table S9) with amino acid identities ranging from 30% to 85% compared to the R. flavefaciens sequences (Table S11). Three sequences encoded scaffoldins with dockerin modules, which may allow integration with additional scaffoldins and multiple enzymes to form the cellulosomal complex.

Besides the genomic prediction analysis, the ability of *Ruminococcus* ERACg_42 to produce cellulosomes was supported by proteomic analysis. Among the proteins secreted by *Ruminococcus* ERACg_42, 37 from 52 predicted cellulosomal proteins were detected, including 8 putative scaffoldins, 1 mixed cellulase-xylanase, 1 β-lactosidase, 1 carboxylesterase, 2 pectinases, 11 cellulases, and 13 hemicellulases appended to dockerin modules (Tables 3 and 4; Tables S9 and S10), accounting for 521 of the total spectrum counts. Although the number of hemicellulases detected was slightly higher than the number of cellulases detected, the total spectrum counts for cellulases was 205, whereas 111 were counted for hemicellulases (Table 4). Therefore, cellulosomes derived from *Ruminococcus* ERACg_42 cells grown on sugarcane bagasse showed a profile that was predominantly cellulolytic, followed by hemicellulolytic and pectinolytic. Moreover, among the cellulases predicted from the *Ruminococcus* sp. ERACg_42 draft genome, only endoglucanases were detected in the metaproteome.

The *Ruminococcus* type strains with ERACg_42 harbored 30 different GH families involved in lignocellulosic degradation, while the closely related species R. flavefaciens ATCC 19208 encodes 28 GH families. The *Ruminococcus* strain harboring ERACg_42 represents the third *Ruminococcus* species described to produce cellulosomes, since only R. flavefaciens ATCC 19208 and R. champanellensis JCM 17042 are known to produce cellulosomes (45, 49, 50).

## DISCUSSION

Some previous studies have reported the enrichment of microbial consortia using different carbon sources, inocula, and culture conditions (9, 19, 23–25, 28, 29, 31, 54). The resulting consortia are frequently described to have observed shifts in microbial communities in response to the carbon source used during the enrichment process. Even though these consortia have been shown to possess lignocellulolytic capabilities, genome-centric investigations and metaproteome analyses of these microbial communities have been barely exploited to date. Therefore, enriched microbial communities require a more comprehensive and deeper analysis of their genetic content and protein production capabilities, to provide novel insights into the syntrophic interaction among the lignocellulolytic members of the consortium.

To address this knowledge gap, we combined several approaches to exploit the lignocellulolytic capabilities of the ERAC. The consortium was established on sugarcane bagasse using as an inoculum source the rumen sample from a fistulated cow which was grazing on natural pastures. The first assessment of the lignocellulolytic capability of the ERAC indicated enzymatic activities against different polysaccharides, followed by modification on bagasse fibers, visualized by SEM. Based on these results, we combined taxonomic profiling, metagenomics, and metaproteomics approaches to evaluate the microbial structure and the enzymatic machinery associated with lignocellulose degradation present in the ERAC.

The 16S rRNA amplicon analyses showed that the diversity was significantly lower in the ERAC than in the rumen inoculum sample (Fig. 3; see also Table S3 and Fig. S2 in the supplemental material). During the enrichment process, it has been observed that microorganisms with a metabolic function compatible with the cultivation conditions employed are selected and become dominant (9, 25, 26, 55–57). Decreasing diversity, for example, the consortium target for the degradation of quinoline (57), lignin (26), phenanthrene (55), and keratins (56), as well as the reduction of heavy metal (58), was also shown by other studies. Here, the ERAC was dominated by *Firmicutes* and *Bacteroidetes*, which are reported to be degraders of lignocellulosic biomass in several anaerobic environments, such as biogas reactors (59), landfill (60), and insect gut (9). Both phylogenetic groups are well-known to contain an extensive repertoire of CAZymes and enzymatic complexes (6, 7, 13).

For a deeper exploitation of the metagenome data, gene- and genome-centric metagenome analyses were carried out. The gene-centric analysis provided an overview of the entire metabolic potential of the ERAC. The resulting data identified a high proportion of genes associated with carbohydrate and amino acid metabolism (Fig. S3 and S4). These findings are consistent with the fact that the microbial community was enriched on lignocellulose biomass, where genes of carbohydrate metabolism should be highly abundant. Moreover, several conserved protein domain sequences related to lignin degradation were identified in this strictly anaerobic consortium. Although previous studies reported lignin degradation under anaerobic conditions (61–63), the mechanisms of decomposition are still poorly understood; thus, further analyses are required.

Based on genome-centric metagenome analysis, we were able to reconstruct 41 enriched rumen anaerobic consortium genomes (ERACgs) belonging to five phyla. The high level of completeness of the ERACgs allowed a detailed determination of potential degraders in this enriched anaerobic consortium as well as whether they harbor genes to produce enzymatic complexes. Among the ERACgs, those assigned to *Firmicutes* and *Bacteroidia* were predominant and harbored the highest number and diversity of CAZymes. Moreover, all *Bacteroidia* ERACgs and a *Clostridia* ERACg (ERACg_42) were identified to be able to produce PULs and cellulosomes, respectively. Interestingly, ERACgs encoding PULs were identified to have genes encoding cellulolytic enzymes (from the GH5 and GH9 families). Although *Prevotella* species have been reported to use several polysaccharides as sole carbon sources (64, 65), there is no experimental evidence of cellulose depolymerization by PULs (66).

According to our phylogenetic analysis, the isolate with ERACg_42 can confidently be assigned as a species of the *Ruminococcus* genus, closely related to R. flavefaciens ATCC 1920. ERACg_42 encodes a repertoire of cellulosomal proteins and enzymes appended to dockerin modules, making the strain with this genome a potential cellulosome producer. The *Ruminococcus* ERACg_42, however, possesses scaffoldin proteins with the lowest identity to protein sequences available in the public database. We also carried out additional sequence analysis in an attempt to classify the scaffoldins according to the terminology proposed by Brás et al. (67). However, as the scaffoldin sequences of *Ruminococcus* sp. ERACg_42 share a low degree of identity with the corresponding homologous sequences of R. flavefaciens ATCC 19208 (Table S11), it was not possible to confidently classify scaffoldins from *Ruminococcus* sp. ERACg_42. Further experimental investigation must be carried out to determine their classification. Furthermore, differently from the R. flavefaciens ATCC 19208 cellulosomes, which are mostly composed of type III dockerin- and cohesin-containing proteins (68, 69), *Ruminococcus* sp. ERACg_42 encodes the majority of the cellulosomal proteins and CAZymes appended to type I dockerin and cohesin proteins. The type I and type II cohesin modules are frequently found in C. thermocellum and other cellulosome-producing clostridia (44, 49, 70, 71). The unconventional arrangements of the types of cohesin-dockerin modules, which have not been previously reported in this phylotype, in addition to unclassified scaffoldins, might represent novel architectural and functional aspects of cellulosomes.

In this study, *Firmicutes* and *Bacteroidetes* ERACgs were abundantly identified, and these organisms might be the major players responsible for synergistically acting to degrade sugarcane bagasse in this anaerobic consortium. Indeed, metaproteome analysis detected several cellulosomal proteins and a diverse set of CAZymes secreted by the *Ruminococcus* ERACg_42, including the production of cellulosomes with structures similar to those reported previously (49, 53, 69). Components of PULs (*Bacteroidetes*), such as SusCD proteins, were also detected, suggesting that another type of enzymatic complex is also produced.

Our multi-omics study disclosed secreted CAZymes, cellulosomes, PULs, and several nearly complete genomes from anaerobic lignocellulolytic microbes. The ERAC harbored the highest number of CAZymes when the number was compared to the number found in previously characterized anaerobic consortia (23) (Table S12). Compared to three other composting-derived consortium studies established under static conditions (19, 24, 72), the ERAC is the second in terms of total CAZyme number (Table S12). The ERAC also presented the second highest diversity of families in the CAZy database (Table S13) compared to that found in similar previous studies (6, 19, 24, 72). The apple pomace-adapted compost microbial community (72) mapped 13 additional families in the CAZy database (and two GH other families) compared to ERAC. However, the former study (72) examined 64% more protein-coding sequences than the present study (Table S13).

In conclusion, the integrative analysis incorporating metagenomic and metaproteomic approaches reported here has been shown to be a practical guide and a powerful strategy. This discovery approach extends the number of novel CAZymes, enzymatic complexes, and the respective microorganisms producing them, representing results beyond the current knowledge from the enrichment process. The vast and diverse reservoir of new CAZyme sequences discovered here opens up further avenues of opportunity, such as biochemical and structural studies of novel lignocellulolytic enzyme candidates. In addition, the enzymatic complexes reported here are composed of new sequences and may be applied to design artificial enzymatic complexes for future biotechnological applications.

## MATERIALS AND METHODS

### Rumen-derived anaerobic consortium design.

An ERAC was established using cow rumen samples and sugarcane bagasse (SB) (see Table S14 in the supplemental material) as microbial and carbon sources, respectively. Fresh rumen samples (approximately 20 g) were taken from a fistulated cow which was grazing on natural pastures prior to the experimental period at the farm of the Department of Ruminants at the Luiz de Queiroz College of Agriculture (ESALQ/USP, Piracicaba, Brazil). Subsequently, the samples were immediately placed into a prewarmed thermos flask as a means to transport them to the laboratory. The rumen samples were kindly provided by the Department of Ruminants at the Luiz de Queiroz College of Agriculture (ESALQ/USP, Piracicaba, Brazil). All procedures related to animal experiments were undertaken following the guidelines of the Committee on Ethics in the Use of Animals (CEUA) of the Luiz de Queiroz College of Agriculture.

The rumen content was homogenized and mixed (1:4) with prewarmed anaerobic McDougall buffer (39°C) (73) inside an anaerobic chamber (Whitley DG250 anaerobic workstation) under 10% H_2_, 5% CO_2_, and 85% N_2_. Aliquots (2 ml) from mixed solutions were inoculated into 100-ml serum bottles containing 48 ml of growth medium supplemented with 500 mg of sterilized SB, which had previously been wrapped in aluminum foil and sterilized by autoclaving. Then, aliquots (1 ml) of the microbial suspension were transferred under strict anaerobic conditions to fresh medium every 5 days for 25 consecutive passages. The growth medium was prepared as described previously (72). Briefly, the medium was deoxygenated by gassing CO_2_ and dispensed anaerobically in serum bottles inside an anaerobic chamber. The bottles were closed with a stopper, sealed, and autoclaved. Aliquots of 500 mg sterilized SB were added to the bottles, and the bottles were then reclosed and incubated under anoxic conditions. The biological experiments were performed in triplicate, and the bottles were incubated at 39°C under constant conditions.

### Total microbial DNA isolation.

Microbial DNA was extracted from the anaerobic consortium as described previously (74), with modifications. Briefly, an aliquot of a biological replicate from the ERAC culture was centrifuged at 12,000 × *g* for 20 min at 4°C. The resulting pellet was suspended in lysis buffer (100 mM EDTA, 50 mM NaCl, 10 mM Tris, pH 8, 1% SDS, proteinase K). The mixture was incubated at 37°C for 1 h with shaking. To ensure cell lysis, a bead-beating step was carried out using Lysing Matrix E tubes (MP Biomedicals), followed by incubation in a water bath at 65°C for 2 h. After centrifugation, the supernatant was mixed with an equal volume of chloroform-isoamyl alcohol (24:1, vol/vol). The solution was centrifuged, and the aqueous phase was transferred to a clean tube and treated with RNase A (Qiagen, Germantown, MD, USA) for 15 min at 37°C. The DNA was precipitated with isopropanol and resuspended in TE buffer (10 mM Tris-HCl, 1 mM EDTA, pH 8.0). The DNA solution was purified using Power Clean DNA clean-up kits (Mo Bio Laboratories) for the following applications.

### Library preparation.

The V4 region of the 16S rRNA gene was amplified using universal primers (primers 515F and 806R), which cover the *Bacteria* and *Archaea* domains (75). The PCR products obtained were purified with magnetic beads (Beckman Coulter), and the second reaction was carried out on these products to attach multiplex identified (MID) tags between Illumina adapter sequences. The 16S rRNA gene amplicons generated were purified and analyzed using magnetic beads and an Agilent 2100 bioanalyzer system (Agilent), respectively. The purified amplicons were quantified by Kapa Biosystems quantitative PCR (qPCR) library quantification and pooled in equimolar concentrations. The amplicon libraries were constructed in three biological replicates and sequenced on an Illumina MiSeq system (2 × 150 bp), applying the paired-end protocol according to standard procedures.

For metagenomic sequencing purposes, a library was constructed, using a NEBNext Ultra II DNA library preparation kit, by Illumina (New England Biolabs, USA), according to the manufacturer’s instructions. The prepared library was validated and quantified using the Agilent bioanalyzer 2100 system with a 12000 DNA assay kit (Agilent) and a Kapa Biosystems next-generation sequencing library qPCR kit (Kapa Biosystems), respectively. Sequencing was performed using an Illumina HiSeq 2500 platform and applying the paired-end protocol (2 × 150-bp paired ends).

### Sequence data processing and statistical analysis.

The raw 16S rRNA amplicon sequences were preprocessed using a Trimmomatic sequence trimmer (76) to remove the sequencing adapters, low-quality reads (average quality score < 33), and reads with ambiguous bases. Quality-filtered reads were merged by the fast length adjustment of short reads (FLASH) (77) with at least 40 bp of overlap. The unassembled reads were discarded during the merge step. Subsequently, the sequences were analyzed using the QIIME program according to established guidelines reported by Bokulich et al. (78). Briefly, the sequences were compared against the sequences in the Greengenes reference database (79) using the USEARCH program (usearch61 method) to detect chimeric sequences, which were removed. The sequences were clustered into operational taxonomic units (OTUs) using the USEARCH program with a similarity threshold of 97%. Representative sequences of each OTU were aligned by the PyNAST program against the reference database for taxonomic classification via the UCLUST program (EDGAR platform, 2010). To reduce the spurious OTUs, low-abundance OTUs (<0.01% of the sequences) were discarded. The microbial diversity (Shannon and Simpson metrics) and richness (ACE and Chao1 estimators) were calculated in QIIME.

Raw shotgun sequencing data were quality filtered to remove the adapters and reads with a low average quality score as described above. The quality-filtered reads were assembled using the MEGAHIT (v.1.1.1) program (80) with the default settings. The resulting reads were mapped onto the assembled contigs with the Bowtie 2 program (81) to estimate the inclusivity of the metagenome assembly. Analysis of the alignment statistics was performed by the use of SAMtools, which converts the sequence alignment map (SAM) into a binary alignment map (BAM) file and then sorts it. The MetaBAT program (82) was used for the binning process in its very specific mode. Completeness results shown in Table 2 represent the BUSCO 3.0.2 output (92). The completeness and contamination were estimated based on marker genes using the taxonomic workflow of the CheckM (v.1.0.7) program (42). For taxonomic binning, only binned contigs with a completeness of greater than 60% and contamination of less than 10% were assigned to the taxonomic rank using the Phyla-AMPHORA (83) and Kraken (84) tools. Finally, binned contigs were annotated using the Prokka program (85), as described previously (48). Comparative genomic analysis was carried out within the EDGAR platform with the standard settings (52).

### CAZyme, cellulosomal proteins, and PUL prediction.

Searches for CAZymes, scaffolding proteins, and *susCD* gene pairs were performed as previously described (7, 86). Briefly, the amino acid sequences were compared to the sequences in the dbCAN-fam-HMMs database (32), based on hidden Markov models (HMMs), using the HMMER software package (87). The parameters were applied as follows: hits with E values of 1e−6 or not covering 30% of the respective HMM were removed. Predicted sequences in the CAZy database were further compared to the sequences in a custom sequence database derived from the CAZy database using the BLASTp program to determine the percent amino acid sequence identity against those sequences already reported, as described previously (6, 7, 22). To identify potential cellulosomal proteins and PUL, a model cohesin (PF00963), dockerin (PF00404), and SusD-like protein (PF07980) and a model for TonB-dependent receptor/SusC-like proteins (TIGR04056) were downloaded from the Pfam database (https://pfam.xfam.org) and the TIGR-fam database (http://www.tigr.org/TIGRFAMs), respectively, to extend the dbCAN-fam-HMMs database. For PUL prediction, we manually searched for CAZymes predicted within a range of five protein predictions upstream and downstream. The PUL diagrams were drawn using an in-house Python script.

### Liquid chromatography (LC)-MS/MS analysis for metaproteome analysis.

The protein concentration from the supernatant, which was obtained as described previously, was measured using the Bio-Rad protein assay reagent (Bio-Rad Laboratories) according to the Bradford method (88). Bovine serum albumin was used as a standard. Aliquots of 12 μg from the concentrated supernatants were subjected in duplicate to SDS-PAGE using a 12% polyacrylamide gel at 100 V for 1.5 h. The gel was stained by incubating with Coomassie brilliant blue G-250 solution for 3 h on a platform with gentle shaking at room temperature. The gel lanes were cut manually into 12 slices, which were distained with 50% (vol/vol) methanol and 2.5% (vol/vol) acetic acid for 2 h and then dehydrated using acetonitrile. Subsequently, the bands were reduced and alkylated with 10 mM dithiothreitol (DTT) and 50 mM iodoacetamide solutions, respectively, and were then washed with ammonium bicarbonate (for 10 min) and dehydrated and rehydrated using acetonitrile and sodium bicarbonate, respectively. The proteins embedded in the gel slices were digested with trypsin (Promega Corp., Madison, WI, USA), dissolved in 100 mM ammonium bicarbonate solution, and incubated at 37°C overnight. The resulting peptides were purified and desalted using self-assembled C_18_ stage tips. The eluted peptides were analyzed on an electron transfer dissociation (ETD)-enabled LTQ Velos Orbitrap mass spectrometer (Thermo Fisher Scientific) coupled with a liquid chromatograph-tandem mass spectrometer (EASY-nLC system; Proxeon Biosystems) through a Proxeon nanoelectrospray ion source. The peptides were separated with 2% to 90% (vol/vol) acetonitrile in 0.1% (vol/vol) formic acid at 0.6 μl/min using a PicoFrit analytical column (20 cm by 75 μm [inside diameter]; particle size, 5 μm; New Objective, Woburn, MA) at a flow rate of 300 nl/min over 27 min. The nanoelectrospray voltage was set to 2.2 kV, and the source temperature was 275°C. The instrument method for the LTQ Velos Orbitrap mass spectrometer was set up in the data-dependent acquisition mode. The full-scan MS spectra (*m/z* 300 to 1,600) were acquired in the Orbitrap analyzer after accumulation to a target value of 1 × 10^6^. The resolution in the Orbitrap mass spectrometer was set to an *r* value of 60,000, and the 20 most intense peptide ions with charge states of ≥2 were sequentially isolated to a target value of 5,000 and fragmented in the linear ion trap by low-energy collision-induced dissociation (CID) (normalized collision energy, 35%). The signal threshold for triggering an MS/MS event was set to 1,000 counts. Dynamic exclusion was enabled with an exclusion size list of 500, an exclusion duration of 60 s, and a repeat count of 1. An activation false-discovery rate (FDR; *q* value) of 0.25 and an activation time of 10 ms were used.

### Metaproteome analysis.

The raw data were converted into a peak list format (.mgf) using the Mascot server (Matrix Science Ltd.). The resulting peaks were searched against the predicted protein sequences from the ERAC metagenome using the Mascot server (Matrix Science). The following search criteria were applied: carbamidomethylation as fixed modifications, oxidation of methionine as a variable modification, one missed trypsin cleavage, and a tolerance of 10 ppm for precursor ions and 1 Da for fragment ions. ScaffoldQ+ software was applied to further analyze the data processed by the Mascot server to validate the MS/MS-based peptide and protein identification. The following parameters were applied: a minimum protein probability of 90%, a minimum peptide probability of 50%, and a unique different minimum peptide of 2. The false-discovery rate (FDR) was adjusted to 1%. Protein quantification was based on the normalized spectrum abundance, which was calculated as the number of spectral counts identifying a protein. The presence of signal peptides and subcellular localization were manually assessed using the signal peptide prediction program SignalP (v.4.0) (89) and the TMHMM (v.2.0) server (90), respectively.

### Enzymatic activity assays.

Enzymatic activity was determined by measuring the amount of reducing sugar released from distinct polysaccharides, including xylan, lichenan, β-glucan, rye arabinoxylan, xyloglucan, rhamnogalacturonan, pectin, mannan and carboxymethyl cellulose sodium salt (CMC). The polysaccharides were purchased from Sigma-Aldrich and Megazyme. All assays were performed using the proteins at a concentration of 100 ng/μl. The enzymatic reactions were performed in a miniaturized fashion by mixing 100 μl of concentrated supernatant, 50 μl of substrate solution (0.5%, wt/vol), and 30 μl of sodium phosphate buffer (0.1 M) at pH 5.5 and incubation at 39°C for 15 min. The reactions were stopped by adding 100 μl of 3,5-dinitrosalicylic acid (DNS), and the mixture was then immediately boiled for 5 min at 99°C (91). The color intensities were measured in an Infinite M200 spectrophotometer (Tecan, Switzerland) at 540 nm. The calibration curves were constructed using glucose, xylose, and mannose as standards. One unit of enzymatic activity corresponds to the amount of enzyme required to release 1 μmol of reducing sugar per minute. All enzymatic activity assays were carried out in biological triplicate.

### Scanning electron microscopy.

The morphology of the sugarcane bagasse samples before and after being used as a carbon source by the anaerobic consortium was examined using scanning electron microscopy (SEM). Samples were mounted over the metal support (stub) with double-sided carbon tape, and a thin layer of gold metal was applied using an automated sputter coater (Bal-Tec, Walluf, Germany) for 1 min. Then, the samples were examined using an FEI Quanta 650 scanning electron microscope (Thermo Fisher Scientific) operating with a 5-kV accelerating voltage. Several images per samples were obtained from different areas to build up two-image databases (for no bagasse degraded and bagasse degraded).

### GC-MS.

The gases produced by the anaerobic microbial consortium were determined in a gas chromatograph (GC 2014 model; Shimadzu) equipped with a thermal conductivity detector (TCD) and a packed column (Shincarbon ST 50/80 mesh). The injector and detector temperatures were set to 200°C. Initially, the temperature of the GC column was 50°C for 3 min, and then it was heated stepwise (5°C/min) until it reached 180°C. Aliquots of 0.5 ml were recovered from the headspace of the serum bottle and injected using nitrogen as the carrier gas.

### Data availability.

The raw sequencing reads of the amplicon, metagenome, and metaproteome were deposited in the GenBank and PRIDE databases under accession numbers PRJEB30762 and PXD019219, respectively. The data sets supporting the conclusions of this article will be provided upon request.

## Supplementary Material

Supplemental file 1

Supplemental file 2

Supplemental file 3

Supplemental file 4

Supplemental file 5

Supplemental file 6

## References

[1] Isikgor FH, Becer CR 2015 Lignocellulosic biomass: a sustainable platform for the production of bio-based chemicals and polymers. Polym Chem 6:4497–4559. doi:10.1039/C5PY00263J.

[2] Meyer AS, Rosgaard L, Sørensen HR 2009 The minimal enzyme cocktail concept for biomass processing. J Cereal Sci 50:337–344. doi:10.1016/j.jcs.2009.01.010.

[3] da Silva Delabona P, Pirota R, Codima CA, Tremacoldi CR, Rodrigues A, Farinas CS 2012 Using Amazon forest fungi and agricultural residues as a strategy to produce cellulolytic enzymes. Biomass Bioenergy 37:243–250. doi:10.1016/j.biombioe.2011.12.006.

[4] Ribeiro DA, Cota J, Alvarez TM, Brüchli F, Bragato J, Pereira BMP, Pauletti BA, Jackson G, Pimenta MTB, Murakami MT, Camassola M, Ruller R, Dillon AJP, Pradella JGC, Paes Leme AF, Squina FM 2012 The Penicillium echinulatum secretome on sugar cane bagasse. PLoS One 7:e50571. doi:10.1371/journal.pone.0050571. 23227186 PMC3515617

[5] Wilhelm RC, Singh R, Eltis LD, Mohn WW 2019 Bacterial contributions to delignification and lignocellulose degradation in forest soils with metagenomic and quantitative stable isotope probing. ISME J 13:413–429. doi:10.1038/s41396-018-0279-6. 30258172 PMC6331573

[6] Svartström O, Alneberg J, Terrapon N, Lombard V, de Bruijn I, Malmsten J, Dalin A-M, Muller EEL, Shah P, Wilmes P, Henrissat B, Aspeborg H, Andersson AF 2017 Ninety-nine de novo assembled genomes from the moose (Alces alces) rumen microbiome provide new insights into microbial plant biomass degradation. ISME J 11:2538–2551. doi:10.1038/ismej.2017.108. 28731473 PMC5648042

[7] Stewart RD, Auffret MD, Warr A, Wiser AH, Press MO, Langford KW, Liachko I, Snelling TJ, Dewhurst RJ, Walker AW, Roehe R, Watson M 2018 Assembly of 913 microbial genomes from metagenomic sequencing of the cow rumen. Nat Commun 9:870. doi:10.1038/s41467-018-03317-6. 29491419 PMC5830445

[8] Stewart RD, Auffret MD, Warr A, Walker AW, Roehe R, Watson M 2019 Compendium of 4,941 rumen metagenome-assembled genomes for rumen microbiome biology and enzyme discovery. Nat Biotechnol 37:953–961. doi:10.1038/s41587-019-0202-3. 31375809 PMC6785717

[9] Auer L, Lazuka A, Sillam-Dussès D, Miambi E, O'Donohue M, Hernandez-Raquet G 2017 Uncovering the potential of termite gut microbiome for lignocellulose bioconversion in anaerobic batch bioreactors. Front Microbiol 8:2623. doi:10.3389/fmicb.2017.02623. 29312279 PMC5744482

[10] Henderson G, Cox F, Ganesh S, Jonker A, Young W, Janssen PH, Abecia L, Angarita E, Aravena P, Arenas GN, Ariza C, Attwood GT, Avila JM, Avila-Stagno J, Bannink A, Barahona R, Batistotti M, Bertelsen MF, Brown-Kav A, Carvajal AM, Cersosimo L, Chaves AV, Church J, Clipson N, Cobos-Peralta MA, Cookson AL, Cravero S, Carballo OC, Crosley K, Cruz G, Cucchi MC, De La Barra R, De Menezes AB, Detmann E, Dieho K, Dijkstra J, Dos Reis WLS, Dugan MER, Ebrahimi SH, Eythórsdóttir E, Fon FN, Fraga M, Franco F, Friedeman C, Fukuma N, Gagić D, Gangnat I, Grilli DJ, Guan LL, Miri VH, 2015 Rumen microbial community composition varies with diet and host, but a core microbiome is found across a wide geographical range. Sci Rep 5:14567. doi:10.1038/srep14567. 26449758 PMC4598811

[11] Denman SE, McSweeney CS 2015 The early impact of genomics and metagenomics on ruminal microbiology. Annu Rev Anim Biosci 3:447–465. doi:10.1146/annurev-animal-022114-110705. 25387109

[12] Seshadri R, Leahy SC, Attwood GT, Teh KH, Lambie SC, Cookson AL, Eloe-Fadrosh EA, Pavlopoulos GA, Hadjithomas M, Varghese NJ, Paez-Espino D, Palevich N, Janssen PH, Ronimus RS, Noel S, Soni P, Reilly K, Atherly T, Ziemer C, Wright A-D, Ishaq S, Cotta M, Thompson S, Crosley K, McKain N, Wallace RJ, Flint HJ, Martin JC, Forster RJ, Gruninger RJ, McAllister T, Gilbert R, Ouwerkerk D, Klieve A, Al Jassim R, Denman S, McSweeney C, Rosewarne C, Koike S, Kobayashi Y, Mitsumori M, Shinkai T, Cravero S, Cucchi MC, Perry R, Henderson G, Creevey CJ, Terrapon N, Lapebie P, Drula E, 2018 Cultivation and sequencing of rumen microbiome members from the Hungate1000 collection. Nat Biotechnol 36:359–367. doi:10.1038/nbt.4110. 29553575 PMC6118326

[13] Gharechahi J, Salekdeh GH 2018 A metagenomic analysis of the camel rumen’s microbiome identifies the major microbes responsible for lignocellulose degradation and fermentation. Biotechnol Biofuels 11:216. doi:10.1186/s13068-018-1214-9. 30083229 PMC6071333

[14] Bule P, Pires VM, Fontes CM, Alves VD 2018 Cellulosome assembly: paradigms are meant to be broken! Curr Opin Struct Biol 49:154–161. doi:10.1016/j.sbi.2018.03.012. 29597100

[15] Bayer EA, Lamed R, White BA, Flint HJ 2008 From cellulosomes to cellulosomics. Chem Rec 8:364–377. doi:10.1002/tcr.20160. 19107866

[16] Grondin JM, Tamura K, Déjean G, Abbott DW, Brumer H 2017 Polysaccharide utilization loci: fuelling microbial communities. J Bacteriol 199:e00860-16. doi:10.1128/JB.00860-16. 28138099 PMC5512228

[17] Lapébie P, Lombard V, Drula E, Terrapon N, Henrissat B 2019 Bacteroidetes use thousands of enzyme combinations to break down glycans. Nat Commun 10:2043. doi:10.1038/s41467-019-10068-5. 31053724 PMC6499787

[18] Creevey CJ, Kelly WJ, Henderson G, Leahy SC 2014 Determining the culturability of the rumen bacterial microbiome. Microb Biotechnol 7:467–479. doi:10.1111/1751-7915.12141. 24986151 PMC4229327

[19] Lemos LN, Pereira RV, Quaggio RB, Martins LF, Moura LMS, da Silva AR, Antunes LP, da Silva AM, Setubal JC 2017 Genome-centric analysis of a thermophilic and cellulolytic bacterial consortium derived from composting. Front Microbiol 8:644. doi:10.3389/fmicb.2017.00644. 28469608 PMC5395642

[20] Campanaro S, Treu L, Kougias PG, Luo G, Angelidaki I 2018 Metagenomic binning reveals the functional roles of core abundant microorganisms in twelve full-scale biogas plants. Water Res 140:123–134. doi:10.1016/j.watres.2018.04.043. 29704757

[21] Kougias PG, Campanaro S, Treu L, Tsapekos P, Armani A, Angelidaki I 2018 Spatial distribution and diverse metabolic functions of lignocellulose-degrading uncultured bacteria as revealed by genomecentric metagenomics. Appl Environ Microbiol 84:e01244-18. doi:10.1128/AEM.01244-18. 30006398 PMC6121989

[22] Snelling TJ, Wallace RJ 2017 The rumen microbial metaproteome as revealed by SDS-PAGE. BMC Microbiol 17:9. doi:10.1186/s12866-016-0917-y. 28061817 PMC5219685

[23] Wong MT, Wang W, Couturier M, Razeq FM, Lombard V, Lapebie P, Edwards EA, Terrapon N, Henrissat B, Master ER 2017 Comparative metagenomics of cellulose- and poplar hydrolysate-degrading microcosms from gut microflora of the Canadian beaver (Castor canadensis) and North American moose (Alces americanus) after long-term enrichment. Front Microbiol 8:2504. doi:10.3389/fmicb.2017.02504. 29326667 PMC5742341

[24] Zhu N, Yang J, Ji L, Liu J, Yang Y, Yuan H 2016 Metagenomic and metaproteomic analyses of a corn stover-adapted microbial consortium EMSD5 reveal its taxonomic and enzymatic basis for degrading lignocellulose. Biotechnol Biofuels 9:243. doi:10.1186/s13068-016-0658-z. 27833656 PMC5103373

[25] Deng Y, Huang Z, Ruan W, Miao H, Shi W, Zhao M 2018 Enriching ruminal polysaccharide-degrading consortia via co-inoculation with methanogenic sludge and microbial mechanisms of acidification across lignocellulose loading gradients. Appl Microbiol Biotechnol 102:3819–3830. doi:10.1007/s00253-018-8877-9. 29511848

[26] Moraes EC, Alvarez TM, Persinoti GF, Tomazetto G, Brenelli LB, Paixão DAA, Ematsu GC, Aricetti JA, Caldana C, Dixon N, Bugg TDH, Squina FM 2018 Lignolytic-consortium omics analyses reveal novel genomes and pathways involved in lignin modification and valorization. Biotechnol Biofuels 11:75. doi:10.1186/s13068-018-1073-4. 29588660 PMC5863372

[27] Kolinko S, Wu YW, Tachea F, Denzel E, Hiras J, Gabriel R, Bäcker N, Chan LJG, Eichorst SA, Frey D, Chen Q, Azadi P, Adams PD, Pray TR, Tanjore D, Petzold CJ, Gladden JM, Simmons BA, Singer SW 2018 A bacterial pioneer produces cellulase complexes that persist through community succession. Nat Microbiol 3:99–107. doi:10.1038/s41564-017-0052-z. 29109478 PMC6794216

[28] Wong MT, Wang W, Lacourt M, Couturier M, Edwards EA, Master ER 2016 Substrate-driven convergence of the microbial community in lignocellulose-amended enrichments of gut microflora from the Canadian beaver (Castor canadensis) and North American moose (Alces americanus). Front Microbiol 7:961. doi:10.3389/fmicb.2016.00961. 27446004 PMC4914502

[29] de Lima Brossi MJ, Jiménez DJ, Cortes-Tolalpa L, van Elsas JD 2016 Soil-derived microbial consortia enriched with different plant biomass reveal distinct players acting in lignocellulose degradation. Microb Ecol 71:616–627. doi:10.1007/s00248-015-0683-7. 26487437 PMC4788684

[30] Jiménez DJ, de Lima Brossi MJ, Schückel J, Kračun SK, Willats WGT, van Elsas JD 2016 Characterization of three plant biomass-degrading microbial consortia by metagenomics- and metasecretomics-based approaches. Appl Microbiol Biotechnol 100:10463–10477. doi:10.1007/s00253-016-7713-3. 27418359 PMC5119850

[31] Lazuka A, Auer L, O'Donohue M, Hernandez-Raquet G 2018 Anaerobic lignocellulolytic microbial consortium derived from termite gut: enrichment, lignocellulose degradation and community dynamics. Biotechnol Biofuels 11:284. doi:10.1186/s13068-018-1282-x. 30356893 PMC6191919

[32] Yin Y, Mao X, Yang J, Chen X, Mao F, Xu Y 2012 dbCAN: a web resource for automated carbohydrate-active enzyme annotation. Nucleic Acids Res 40:W445–W451. doi:10.1093/nar/gks479. 22645317 PMC3394287

[33] Cantarel BI, Coutinho PM, Rancurel C, Bernard T, Lombard V, Henrissat B 2009 The Carbohydrate-Active enZymes database (CAZy): an expert resource for glycogenomics. Nucleic Acids Res 37:D233–D238. doi:10.1093/nar/gkn663. 18838391 PMC2686590

[34] Obeng EM, Adam SNN, Budiman C, Ongkudon CM, Maas R, Jose J 2017 Lignocellulases: a review of emerging and developing enzymes, systems, and practices. Bioresour Bioprocess 4:16. doi:10.1186/s40643-017-0146-8.

[35] Zhang H, Yohe T, Huang L, Entwistle S, Wu P, Yang Z, Busk PK, Xu Y, Yin Y 2018 DbCAN2: a meta server for automated carbohydrate-active enzyme annotation. Nucleic Acids Res 46:W95–W101. doi:10.1093/nar/gky418. 29771380 PMC6031026

[36] Machovič M, Janeček Š 2008 Domain evolution in the GH13 pullulanase subfamily with focus on the carbohydrate-binding module family 48. Biologia (Bratisl) 63:1057–1068. doi:10.2478/s11756-008-0162-4.

[37] Fujimoto Z, Jackson A, Michikawa M, Maehara T, Momma M, Henrissat B, Gilbert HJ, Kaneko S 2013 The structure of a Streptomyces avermitilis α-l-rhamnosidase reveals a novel carbohydrate-binding module CBM67 within the six-domain arrangement. J Biol Chem 288:12376–12385. doi:10.1074/jbc.M113.460097. 23486481 PMC3636921

[38] Neumüller KG, Streekstra H, Gruppen H, Schols HA 2014 Trichoderma longibrachiatum acetyl xylan esterase 1 enhances hemicellulolytic preparations to degrade corn silage polysaccharides. Bioresour Technol 163:64–73. doi:10.1016/j.biortech.2014.04.001. 24787318

[39] Moraïs S, Stern J, Kahn A, Galanopoulou AP, Yoav S, Shamshoum M, Smith MA, Hatzinikolaou DG, Arnold FH, Bayer EA 2016 Enhancement of cellulosome-mediated deconstruction of cellulose by improving enzyme thermostability. Biotechnol Biofuels 9:164. doi:10.1186/s13068-016-0577-z. 27493686 PMC4973527

[40] Cheng Y, Wang Y, Li Y, Zhang Y, Liu T, Wang Y, Sharpton TJ, Zhu W 2017 Progressive colonization of bacteria and degradation of rice straw in the rumen by Illumina sequencing. Front Microbiol 8:2165. doi:10.3389/fmicb.2017.02165. 29163444 PMC5681530

[41] Wu S, Zhu Z, Fu L, Niu B, Li W 2011 WebMGA: a customizable web server for fast metagenomic sequence analysis. BMC Genomics 12:444. doi:10.1186/1471-2164-12-444. 21899761 PMC3180703

[42] Parks DH, Imelfort M, Skennerton CT, Hugenholtz P, Tyson GW 2015 CheckM: assessing the quality of microbial genomes recovered from isolates, single cells, and metagenomes. Genome Res 25:1043–1055. doi:10.1101/gr.186072.114. 25977477 PMC4484387

[43] Mackenzie AK, Naas AE, Kracun SK, Schückel J, Fangel JU, Agger JW, Willats WGT, Eijsink VGH, Pope PB 2015 A polysaccharide utilization locus from an uncultured Bacteroidetes phylotype suggests ecological adaptation and substrate versatility. Appl Environ Microbiol 81:187–195. doi:10.1128/AEM.02858-14. 25326301 PMC4272745

[44] Yoav S, Barak Y, Shamshoum M, Borovok I, Lamed R, Dassa B, Hadar Y, Morag E, Bayer EA 2017 How does cellulosome composition influence deconstruction of lignocellulosic substrates in Clostridium (Ruminiclostridium) thermocellum DSM 1313? Biotechnol Biofuels 10:222. doi:10.1186/s13068-017-0909-7. 28932263 PMC5604425

[45] Artzi L, Bayer EA, Moraïs S 2017 Cellulosomes: bacterial nanomachines for dismantling plant polysaccharides. Nat Rev Microbiol 15:83–95. doi:10.1038/nrmicro.2016.164. 27941816

[46] Bensoussan L, Moraïs S, Dassa B, Friedman N, Henrissat B, Lombard V, Bayer EA, Mizrahi I 2017 Broad phylogeny and functionality of cellulosomal components in the bovine rumen microbiome. Environ Microbiol 19:185–197. doi:10.1111/1462-2920.13561. 27712009 PMC6487960

[47] Ben David Y, Dassa B, Borovok I, Lamed R, Koropatkin NM, Martens EC, White BA, Bernalier-Donadille A, Duncan SH, Flint HJ, Bayer EA, Moraïs S 2015 Ruminococcal cellulosome systems from rumen to human. Environ Microbiol 17:3407–3426. doi:10.1111/1462-2920.12868. 25845888

[48] Tomazetto G, Hahnke S, Koeck DE, Wibberg D, Maus I, Pühler A, Klocke M, Schlüter A 2016 Complete genome analysis of Clostridium bornimense strain M2/40T: a new acidogenic Clostridium species isolated from a mesophilic two-phase laboratory-scale biogas reactor. J Biotechnol 232:38–49. doi:10.1016/j.jbiotec.2015.08.001. 26256097

[49] Dassa B, Borovok I, Ruimy-Israeli V, Lamed R, Flint HJ, Duncan SH, Henrissat B, Coutinho P, Morrison M, Mosoni P, Yeoman CJ, White BA, Bayer EA 2014 Rumen cellulosomics: divergent fiber-degrading strategies revealed by comparative genome-wide analysis of six ruminococcal strains. PLoS One 9:e99221. doi:10.1371/journal.pone.0099221. 24992679 PMC4081043

[50] White BA, Lamed R, Bayer EA, Flint HJ 2014 Biomass utilization by gut microbiomes. Annu Rev Microbiol 68:279–296. doi:10.1146/annurev-micro-092412-155618. 25002092

[51] Cann I, Bernardi RC, Mackie RI 2016 Cellulose degradation in the human gut: Ruminococcus champanellensis expands the cellulosome paradigm. Environ Microbiol 18:307–310. doi:10.1111/1462-2920.13152. 26781441

[52] Blom J, Albaum SP, Doppmeier D, Pühler A, Vorhölter F-J, Zakrzewski M, Goesmann A 2009 EDGAR: a software framework for the comparative analysis of prokaryotic genomes. BMC Bioinformatics 10:154. doi:10.1186/1471-2105-10-154. 19457249 PMC2696450

[53] Venditto I, Luis AS, Rydahl M, Schückel J, Fernandes VO, Vidal-Melgosa S, Bule P, Goyal A, Pires VMR, Dourado CG, Ferreira LMA, Coutinho PM, Henrissat B, Knox JP, Baslé A, Najmudin S, Gilbert HJ, Willats WGT, Fontes C 2016 Complexity of the *Ruminococcus flavefaciens* cellulosome reflects an expansion in glycan recognition. Proc Natl Acad Sci U S A 113:7136–7141. doi:10.1073/pnas.1601558113. 27298375 PMC4932953

[54] Carlos C, Fan H, Currie CR 2018 Substrate shift reveals roles for members of bacterial consortia in degradation of plant cell wall polymers. Front Microbiol 9:364. doi:10.3389/fmicb.2018.00364. 29545786 PMC5839234

[55] Jiao S, Chen W, Wang E, Wang J, Liu Z, Li Y, Wei G 2016 Microbial succession in response to pollutants in batch-enrichment culture. Sci Rep 6:21791. doi:10.1038/srep21791. 26905741 PMC4764846

[56] Kang D, Jacquiod S, Herschend J, Wei S, Nesme J, Sørensen SJ 2019 Construction of simplified microbial consortia to degrade recalcitrant materials based on enrichment and dilution-to-extinction cultures. Front Microbiol 10:3010. doi:10.3389/fmicb.2019.03010. 31998278 PMC6968696

[57] Wang Y, Tian H, Huang F, Long W, Zhang Q, Wang J, Zhu Y, Wu X, Chen G, Zhao L, Bakken LR, Frostegård Å, Zhang X 2017 Time-resolved analysis of a denitrifying bacterial community revealed a core microbiome responsible for the anaerobic degradation of quinoline. Sci Rep 7:14778. doi:10.1038/s41598-017-15122-0. 29116183 PMC5677008

[58] Ma L, Xu J, Chen N, Li M, Feng C 2019 Microbial reduction fate of chromium (Cr) in aqueous solution by mixed bacterial consortium. Ecotoxicol Environ Saf 170:763–770. doi:10.1016/j.ecoenv.2018.12.041. 30583287

[59] De Vrieze J, Pinto AJ, Sloan WT, Boon N, Ijaz UZ 2018 The active microbial community more accurately reflects the anaerobic digestion process: 16S rRNA (gene) sequencing as a predictive tool Microbiome 6:63. doi:10.1186/s40168-018-0449-9. 29609653 PMC5879801

[60] Ransom-Jones E, McCarthy AJ, Haldenby S, Doonan J, McDonald JE 2017 Lignocellulose-degrading microbial communities in landfill sites represent a repository of unexplored biomass-degrading diversity. mSphere 2:e00300-17. doi:10.1128/mSphere.00300-17. 28776044 PMC5541161

[61] Ko J, Shimizu Y, Ikeda K, Kim S, Park C, Matsui S 2009 Biodegradation of high molecular weight lignin under sulfate reducing conditions: lignin degradability and degradation by-products. Bioresour Technol 100:1622–1627. doi:10.1016/j.biortech.2008.09.029. 18977138

[62] Deangelis KM, Sharma D, Varney R, Simmons B, Isern NG, Markilllie LM, Nicora C, Norbeck AD, Taylor RC, Aldrich JT, Robinson EW 2013 Evidence supporting dissimilatory and assimilatory lignin degradation in Enterobacter lignolyticus SCF1. Front Microbiol 4:280. doi:10.3389/fmicb.2013.00280. 24065962 PMC3777014

[63] Kato S, Chino K, Kamimura N, Masai E, Yumoto I, Kamagata Y 2015 Methanogenic degradation of lignin-derived monoaromatic compounds by microbial enrichments from rice paddy field soil. Sci Rep 5:14295. doi:10.1038/srep14295. 26399549 PMC4585845

[64] Fehlner-Peach H, Magnabosco C, Raghavan V, Scher JU, Tett A, Cox LM, Gottsegen C, Watters A, Wiltshire-Gordon JD, Segata N, Bonneau R, Littman DR 2019 Distinct polysaccharide utilization profiles of human intestinal Prevotella copri isolates. Cell Host Microbe 26:680–690.e5. doi:10.1016/j.chom.2019.10.013. 31726030 PMC7039456

[65] Vera-Ponce de León A, Jahnes BC, Duan J, Camuy-Vélez LA, Sabree ZL 2020 Cultivable, host-specific Bacteroidetes symbionts exhibit diverse polysaccharolytic strategies. Appl Environ Microbiol 86:e00091-20. doi:10.1128/AEM.00091-20. 32060023 PMC7117922

[66] Solden LM, Naas AE, Roux S, Daly RA, Collins WB, Nicora CD, Purvine SO, Hoyt DW, Schückel J, Jørgensen B, Willats W, Spalinger DE, Firkins JL, Lipton MS, Sullivan MB, Pope PB, Wrighton KC 2018 Interspecies cross-feeding orchestrates carbon degradation in the rumen ecosystem. Nat Microbiol 3:1274–1284. doi:10.1038/s41564-018-0225-4. 30356154 PMC6784887

[67] Brás JLA, Pinheiro BA, Cameron K, Cuskin F, Viegas A, Najmudin S, Bule P, Pires VMR, Romaõ MJ, Bayer EA, Spencer HL, Smith S, Gilbert HJ, Alves VD, Carvalho AL, Fontes C 2016 Diverse specificity of cellulosome attachment to the bacterial cell surface. Sci Rep 6:38292. doi:10.1038/srep38292. 27924829 PMC5141474

[68] Dai X, Tian Y, Li J, Su X, Wang X, Zhao S, Liu L, Luo Y, Liu D, Zheng H, Wang J, Dong Z, Hu S, Huang L 2015 Metatranscriptomic analyses of plant cell wall polysaccharide degradation by microorganisms in the cow rumen. Appl Environ Microbiol 81:1375–1386. doi:10.1128/AEM.03682-14. 25501482 PMC4309707

[69] Israeli-Ruimy V, Bule P, Jindou S, Dassa B, Morais S, Borovok I, Barak Y, Slutzki M, Hamberg Y, Cardoso V, Alves VD, Najmudin S, White BA, Flint HJ, Gilbert HJ, Lamed R, Fontes C, Bayer EA 2017 Complexity of the Ruminococcus flavefaciens FD-1 cellulosome reflects an expansion of family-related protein-protein interactions. Sci Rep 7:42355. doi:10.1038/srep42355. 28186207 PMC5301203

[70] Raman B, Pan C, Hurst GB, Rodriguez M, McKeown CK, Lankford PK, Samatova NF, Mielenz JR 2009 Impact of pretreated switchgrass and biomass carbohydrates on Clostridium thermocellum ATCC 27405 cellulosome composition: a quantitative proteomic analysis. PLoS One 4:e5271. doi:10.1371/journal.pone.0005271. 19384422 PMC2668762

[71] Phitsuwan P, Moraïs S, Dassa B, Henrissat B, Bayer EA 2019 The cellulosome paradigm in an extreme alkaline environment. Microorganisms 7:347. doi:10.3390/microorganisms7090347. PMC6780208 31547347

[72] Couger MB, Youssef NH, Struchtemeyer CG, Liggenstoffer AS, Elshahed MS 2015 Transcriptomic analysis of lignocellulosic biomass degradation by the anaerobic fungal isolate Orpinomyces sp. strain C1A. Biotechnol Biofuels 8:208. doi:10.1186/s13068-015-0390-0. 26649073 PMC4672494

[73] McDougall EI 1948 Studies on ruminant saliva. 1. The composition and output of sheep’s saliva. Biochem J 43:99–109. doi:10.1042/bj0430099. PMC1274641 16748377

[74] Tomazetto G, Wibberg D, Schlüter A, Oliveira VM 2015 New FeFe-hydrogenase genes identified in a metagenomic fosmid library from a municipal wastewater treatment plant as revealed by high-throughput sequencing. Res Microbiol 166:9–19. doi:10.1016/j.resmic.2014.11.002. 25446611

[75] Caporaso JG, Lauber CL, Walters WA, Berg-Lyons D, Lozupone CA, Turnbaugh PJ, Fierer N, Knight R 2011 Global patterns of 16S rRNA diversity at a depth of millions of sequences per sample. Proc Natl Acad Sci U S A 108:4516–4522. doi:10.1073/pnas.1000080107. 20534432 PMC3063599

[76] Bolger AM, Lohse M, Usadel B 2014 Trimmomatic: a flexible trimmer for Illumina sequence data. Bioinformatics 30:2114–2120. doi:10.1093/bioinformatics/btu170. 24695404 PMC4103590

[77] Magoc T, Salzberg SL 2011 FLASH: fast length adjustment of short reads to improve genome assemblies. Bioinformatics 27:2957–2963. doi:10.1093/bioinformatics/btr507. 21903629 PMC3198573

[78] Bokulich NA, Subramanian S, Faith JJ, Gevers D, Gordon JI, Knight R, Mills DA, Caporaso JG 2013 Quality-filtering vastly improves diversity estimates from Illumina amplicon sequencing. Nat Methods 10:57–59. doi:10.1038/nmeth.2276. 23202435 PMC3531572

[79] McDonald D, Price MN, Goodrich J, Nawrocki EP, DeSantis TZ, Probst A, Andersen GL, Knight R, Hugenholtz P 2012 An improved Greengenes taxonomy with explicit ranks for ecological and evolutionary analyses of bacteria and archaea. ISME J 6:610–618. doi:10.1038/ismej.2011.139. 22134646 PMC3280142

[80] Li D, Luo R, Liu CM, Leung CM, Ting HF, Sadakane K, Yamashita H, Lam TW 2016 MEGAHIT v1.0: a fast and scalable metagenome assembler driven by advanced methodologies and community practices. Methods 102:3–11. doi:10.1016/j.ymeth.2016.02.020. 27012178

[81] Langmead B, Salzberg SL 2012 Fast gapped-read alignment with Bowtie 2. Nat Methods 9:357–359. doi:10.1038/nmeth.1923. 22388286 PMC3322381

[82] Kang DD, Froula J, Egan R, Wang Z 2015 MetaBAT, an efficient tool for accurately reconstructing single genomes from complex microbial communities. PeerJ 3:e1165. doi:10.7717/peerj.1165. 26336640 PMC4556158

[83] Wang Z, Wu M 2013 A phylum-level bacterial phylogenetic marker database. Mol Biol Evol 30:1258–1262. doi:10.1093/molbev/mst059. 23519313

[84] Wood DE, Salzberg SL 2014 Kraken: ultrafast metagenomic sequence classification using exact alignments. Genome Biol 15:R46. doi:10.1186/gb-2014-15-3-r46. 24580807 PMC4053813

[85] Seemann T 2014 Prokka: rapid prokaryotic genome annotation. Bioinformatics 30:2068–2069. doi:10.1093/bioinformatics/btu153. 24642063

[86] Tomazetto G, Hahnke S, Wibberg D, Pühler A, Klocke M, Schlüter A 2018 Proteiniphilum saccharofermentans str. M3/6T isolated from a laboratory biogas reactor is versatile in polysaccharide and oligopeptide utilization as deduced from genome-based metabolic reconstructions. Biotechnol Rep (Amst) 18:e00254. doi:10.1016/j.btre.2018.e00254. 29892569 PMC5993710

[87] Eddy SR 1998 Profile hidden Markov models. Bioinformatics 14:755–763. doi:10.1093/bioinformatics/14.9.755. 9918945

[88] Bradford MM 1976 A rapid and sensitive method for the quantitation of microgram quantities of protein utilizing the principle of protein-dye binding. Anal Biochem 72:248–254. doi:10.1006/abio.1976.9999. 942051

[89] Petersen TN, Brunak S, Von Heijne G, Nielsen H 2011 SignalP 4.0: discriminating signal peptides from transmembrane regions. Nat Methods 8:785–786. doi:10.1038/nmeth.1701. 21959131

[90] Krogh A, Larsson B, von Heijne G, Sonnhammer EL 2001 Predicting transmembrane protein topology with a hidden Markov model: application to complete genomes. J Mol Biol 305:567–580. doi:10.1006/jmbi.2000.4315. 11152613

[91] Miller GL 1959 Use of dinitrosaiicyiic acid reagent for determination of reducing sugar. Anal Chem 31:426–428. doi:10.1021/ac60147a030.

[92] Simão FA, Waterhouse RM, Ioannidis P, Kriventseva EV, Zdobnov EM 2015 BUSCO: assessing genome assembly and annotation completeness with single-copy orthologs. Bioinformatics 31:3210–3212. doi:10.1093/bioinformatics/btv351. 26059717

