# Supplementary material for: Multi-omic Directed Discovery of Cellulosomes, Polysaccharide Utilization Loci, and Lignocellulases from an Enriched Rumen Anaerobic Consortium
Source: Appl Environ Microbiol. 2020 Sep 1;86(18):e00199-20. doi: 10.1128/AEM.00199-20 (PMC7480376; doi:10.1128/AEM.00199-20)
Supplement: Supplemental file 1 [file AEM.00199-20-s0001.pdf]

Multi-omic directed discovery of cellulosomes, PULs and lignocellulases from an enriched rumen anaerobic consortium

Geizecler Tomazetto[1,2], Agnes C. Pimentel[2], Daniel Wibberg[3], Neil Dixon[2], Fabio M Squina[1*]

[1] Programa de Processos Tecnológicos e Ambientais, Universidade de Sorocaba, Sorocaba, Brasil.

[2] Manchester Institute of Biotechnology, Department of Chemistry, University of Manchester, Manchester, United Kingdom.

[3] Departamento de Bioquímica, Instituto de Biologia (IB), Universidade Estadual de Campinas (UNICAMP), - Cidade Universitária, Campinas, São Paulo, Brasil.

[4] Center for Biotechnology (CeBiTec), Genome Research of Industrial Microorganisms, Bielefeld University, Bielefeld, Germany.

Correspondence to fabio.squina@prof.uniso.br; Rod. Raposo Tavares, km, 92.5, Sorocaba-SP, CEP 18023-000, Brasil

This PDF file includes:

Additional Result and Discussion section

Tables S1 to S14

Figs. S1 to S4

References

Result and Discussion

CAZyme profile of the enriched rumen anaerobic consortium

The ERAC metagenome contains 2,158 glycoside hydrolases (GH), 695 carbohydrate-binding modules (CBM), 17 cohesin, 159 dockerin, 1,457 glycosyltransferases (GT), 858 carbohydrate esterases (CE), 69 polysaccharide lyases (PL), 176 auxiliary activities (AAs), and 175 S-layer homology (SLH) modules. A summarization overview of all predicted CAZy families is described in Table 1 and Additional file 2. The complete analysis about GH families is found in the article.

Analyzing in more detail CAZyme prediction, the ERAC contains 92 distinct GH families (Additional file 3). Among them, we found GH families encoding cellulases (GH5, GH8, GH9, GH44, GH48 and GH124), oligosaccharide-degrading enzymes (GH1, GH2, GH3, GH29, GH35, GH38, GH39, GH42 and GH94), mannases (GH76, GH92 and GH125), pectinases (GH28, GH78, and GH93), chitinases (GH18 and GH19), α-amylases (GH13, GH57, GH77, and GH126), and xylanases (GH10, GH11, GH30, GH43, and GH127).

The remaining CAZyme families, such as CE, PL, and AA, also play important role in lignocellulose breakdown, boosting the hydrolysis of lignocellulosic biomassSeveral different families of CEs, PLs, and AAs were also contained within the CAZyme repertoire from ERAC. Carbohydrate esterases (CEs) remove ester bonds from mono-, oligo-, and polysaccharides, facilitating the action of GHs on complex polysaccharides. The ERAC presented a wide spectrum of CE families, 14 out of the 16 defined CE families were encountered. The families CE1 and CE4, characterized by their member's ability to deacetylate polymeric xylans (1, 2), were the most prevalent ones present in the consortium. Additional families related with xylan degradation, such as CE2, CE3, CE6, and CE7, were also predicted. Moreover, CE8, CE12, CE13 and CE15 families that encode enzymes for pectin, rhamnogalacturanan, xylan, glucuronoxylans, and alginates degradation (3, 4), respectively, were also found (Additional file 2).

PLs families encode enzymes that cleave glycosidic linkages present in uronic acid-containing polysaccharides (e.g. alginate, pectin, and xanthan). Although only a small

fraction of PLs sequences in the anaerobic consortium were predicted as PLs, 12 out of 19 PLs families (defined by CAZy) were encountered (Table 1 and Additional file 2). Among them, families encoding enzymes involved in the degradation of oligo-galacturonate lyase (PL12 and PL22), pectin (PL1 and PL9), alginate (PL5 and PL15), and xanthan (PL8).

The auxiliary activity families contain oxidative ligninolytic enzymes and lytic polysaccharide monoxygenases. The AA families were categorized into five families, of which AA6 (1,4-benzoquinone reductase) was the most prevalent. Even though most microorganisms identified in this consortium are strictly anaerobic, the prediction of oxidative AA families was also previously observed from metagenomic anaerobic samples, including camel rumen camel (5) and landfill (6).

Non-GH CAZy members, such as CBMs, are protein domains found in carbohydrate-active enzymes that can potentiate the activity of the associate catalytic domains (7). The set of predicted CBMs ERAC comprised 43 families, of which surprisingly CBM50, predicted to bind peptidoglycan-like and chitin-derived oligosaccharides (8), was the most prevalent encountered in the metagenome dataset (Table 1). The CBM50 were predominantly predicted in association to GH23 and GH73, both families related with peptidoglycan degradation. Similar abundance was also found in the moose rumen microbiome (9) and camel rumen (5). In our study, genes encoding CBMs that bind on xylan (CBM4, CBM9, CBM16, CBM22, CBM35, and CBM36), cellulose (CBM2, CBM3, CBM6, CBM11, CBM13, CBM30, and CBM63), starch (CBM20, CBM26, CBM34, and CBM69), pullulan (CBM41 and CBM68), and glucans (CBM 56, CBM65, CBM 76, CBM 79, and CBM 80) were predicted (Table 1 and Additional file 2). Overall, the ERAC is composed of microorganisms encoding a wide variety of carbohydrate degrading genes with the potential to produce a broad range of enzymatic activities for deconstructing the whole component plant cell wall.

Comparative analysis of ERAC with other enrichment strategies

We compared ERAC and similar consortia also targeting biomass degradation (Table S12). The consortia used for comparative analysis were moose rumen- and beaver dropping-derived anaerobic consortia (4), compost-derived consortia (EMSD5) (10),

bacterial consortium derived from composting (ZCTH02) (11) and apple pomace-adapted compost microbial community (APACMC, (12), established under static condition. Although studies of enrichment strategies have been reported, to our knowledge, our study is the first to apply multi-omics strategies (and other comprehensive analyses), to study anaerobic-enriched microbial community. In addition, our study is unique as it reveals the secreted CAZymes, cellulosomes, PULs, as well several nearly complete genomes from lignocellulolytic microbes. For example, the ZCTH02 consortium reported six near-complete reconstructed genomes (11) (Table S12). The moose rumen- and beaver dropping-derived anaerobic consortia reported dockerin- or cohesin-containing proteins and PULs (4), nonetheless, the presence of cellulosomes in metagenomic data is unclear.

The ERAC harbored the highest diversity and abundance of CAZymes compared to the other strictly anaerobic consortia (Table S12). In addition, it displayed a more complete CAZyme profile related to lignocellulose breakdown compared to previous consortia developed in anoxic conditions. The ERAC was enriched with sequences from families CE1, GH13, CE10, CE4, GH3, GH2, GH43 and GH5 (Additional file 2), whereas the moose rumen- and dropping-derived anaerobic consortia were enriched with a higher proportion of GH3, GH2, GH5, GH43, CE1 and GH53 (4). From previous studies it was concluded that there are nine GH families exclusive in ERAC, the families encoding endo-β-1,4-glucanase from GH124 family, β-1,3-1,4 glucanases from GH64, endo-α-1,4-polygalactosaminidase GH144, arabinofuranosidases GH1237 and rhamnohydrolases (GH145).

Furthermore, when compared to the three composting-derived consortia established under static condition (APACMC, ZCTH02, and EMSD5), ERAC is the second in terms of CAZyme abundance numbers and diversity (Table S12). The ERAC encodes approximately half of CDS (142,703) compared to APACMC (220,767), which encode 9,274 CAZymes. Our anaerobic consortium harbors 5,766 CAZymes distributed among 92 distinct GH families, followed by 43 CBM, 14 CE, 12 PL, and 5 AA families. Whereas the consortium APACMC encodes 94 distinct GH families, followed by 16 CE, 15 PL, and 7 AA families.

Table S1. Percentage of gases detected by GC-MS analysis in the enriched rumen anaerobic consortium (ERAC) evaluated during the seven last passages of the enrichment process.

**Passages** [1]	**% H_2**	**% O_2**	**% CH_4**	**% CO_2**
25th passage	8,08	0,00	0,00	91,91
24th passage	4,71	0,00	0,00	95,29
23th passage	7,07	0,00	0,00	92,92
22th passage	1,19	0,00	0,00	98,80
21th passage	5,66	0,00	0,00	94,33
20th passage	6,25	0,00	0,00	93,74
19th passage	9,19	0,00	0,00	90,80

[1] Gases were collected at the end of every enrichment cycle during the last seven passages. The samples collected were immediately injected into a GC-MS.

Table S2. Sequencing statistics and data processing of 16S rRNA gene amplicon libraries constructed for profiling of the cow rumen (CR) and enriched rumen anaerobic consortium (ERAC) samples.

Samples/Gene amplicon/Replicate	Raw data[1]	Sequences Trimming/Merged/Chimeras[2]	Average amplicon size [pb]	OTUs[3] > 0.01%[4]
CR/16S rRNA/ A	150,576	27,871	289.4	707
CR/16S rRNA/ B	172,104	17,642	289.4	696
ERAC/16S rRNA/ A	78,225	49,38	289.4	269
ERAC /16S rRNA/ B	99,523	53,166	289.4	279
ERAC /16S rRNA/ C	103,592	60,847	289.4	298

[1] Total counting of sequences reads

[2] After quality filtering reads, merged sequences and removal of chimeric sequences.

[3] Operational Taxonomic Units (OTUs)

[4] OTUs featuring reads counts of less than 0.01 % of the total reads were removed from OUT table.

Table S3. Richness and diversity indices of cow rumen (CR) and rumen anaerobic consortium (ERAC) samples based on 16S rRNA gene amplicon sequencing.

Sample/Replicate	Chao1[1]	ACE[1]	Shannon[2]	Simpson[2]
CR/ A	717	710	8.13	0.99
CR /B	704	700	8.1	0.99
ERAC/A	313	305	3.7	0.79
ERAC/B	287	283	3.7	0.79
ERAC/C	277	273	3.8	0.8

[1] Chao1 and ACE are richness indices.

[2] Simpson and Shannon are diversity indices.

Table S4. Pfam domains related with lignin and aromatic degradation found in enriched rumen anaerobic consortium genomes (ERACgs) metagenome data

**Aromatic compound pathway**	**MAG[1]**	**Protein_ID**	**Pfam accession**	**Description**
Bacterial β-ketoadipate pathway (ortho-cleavage)	ERACg_2	k141_68259_110	PF00108	Beta-ketoadipyl-CoA thiolase
	ERACg_32	k141_37736_340	PF00561	3-oxoadipate enol-lactonase
	ERACg_13	k141_76557_21	PF01144	3-oxoadipate enol-transferase
	----	not predicted	PF02803	Beta-ketoadipyl-CoA thiolase
Bacterial β-ketoadipate pathway (ortho-cleavage) Catechol	ERACg_36	k141_7880_6	PF02746	Muconate cycloisomerase
	----	not predicted	PF13378	Enolase C-terminal domain-like
	----	not predicted	PF00775	catechol 1,2-dioxygenase
	----	not predicted	PF02426	Muconolactone isomerase
	----	not predicted	PF04444	catechol 1,2-dioxygenase
Bacterial β-ketoadipate pathway (ortho-cleavage) Protocatechuate	ERACg_59	k141_30916_162	PF00206	4-carboxy-cis,cis -muconate cycloisomerase
	ERACg_57	k141_64819_26	PF02627	Carboxymuconolactone decarboxylase
	ERACg_41	k141_19170_318	PF10397	3-carboxy - cis,cis -muconate cycloisomerase
	----	not predicted	PF00775	Protocatechuate 3,4 -dioxygenase alpha chain
	----	not predicted	PF12391	Protocatechuate 3,4 -dioxygenase beta chain
Bacterial catabolic pathways for the degradation of ferulic acid	ERACg_13	k141_76557_20	PF00378	Feruloyl -CoA hydratase/lyase
	ERACg_49	k141_24753_8	PF13380	Feruloyl -CoA synthetase
	ERAC_38	k141_33477_179	PF13607	Feruloyl -CoA synthetase
β-aryl ether degradation	ERACg_57	k141_93885_6	PF00106	NAD depend dehydrogenase
	ERACg_9	k141_66624_5	PF00171	Vanillin dehydrogenase
	ERACg_46	k141_69205_19	PF01571	Vanillate/3-O-methylgallate O-demethylase

	ERACg_34	k141_98597_25	PF13417	Beta-etherases and glutathione S-transferase
	----	not predicted	PF08669	Vanillate/3-O-methylgallate O-demethylase
	----	not predicted	PF00043	Beta-etherase
	----	not predicted	PF02798	Beta-etherase
Biphenyl component degradation	ERACg_4	k141_96936_3	PF00355	non-heme iron-dependent demethylase enzyme
	ERACg_31	k141_58207_129	PF02900	Extradiol dioxygenase
	ERACg_37	k141_32530_326	PF04909	5-carboxyvanillate decarboxylase
Meta-Cleavage	ERACg_38	k141_11035_9	PF01408	4-carboxy-2-hydroxymuconate-6-semialdehyde dehydrogenase
	ERACg_15	k141_100405_9	PF03737	4-carboxy-4-hydroxy-2-oxoadipate aldolase
	----	not predicted	PF02900	Protocatechuate 4,5-dioxygenase beta chain
	----	not predicted	PF04909	2-pyrone-4,6-dicarbaxylate hydrolase
	----	not predicted	PF07746	Protocatechuate 4,5-dioxygenase alpha chain
Oxidative degradation	dataset[2]	k141_23274_1	PF00141	Peroxidase
	dataset	k141_35969_1	PF00394	Multicopper oxidase
	ERACg_49	k141_43427_7	PF02578	Multi-copper polyphenol oxidoreductase laccase
	dataset	k141_35969_1	PF07731	Multicopper oxidase
	ERACg_56	k141_68029_26	PF06537	Di-haem oxidoreductase, putative peroxidase
	ERACg_54	k141_91874_43	PF00199	Catalase
	----	not predicted	PF04261	Dye-decolorizing peroxidase
	----	not predicted	PF07732	Laccase

[1] Metagenome-assembly genomes (MAGs)

[2] Sequences coding proteins were associated with MAGs.

Table S5 Overview of the multi-domain organizations for CBMs modules predicted in enriched rumen anaerobic consortium (ERAC)

Domain organization	Number	Multi-Domain	Number
CBM48-GH13_9	26	CBM38-GH116	1
CBM67-GH78	24	CBM38-GH32	1
CBM34-GH13_20	11	CBM4-CBM4-GH16-CBM4-CBM4	1
CBM48-CE1	9	CBM4-GH51	1
GH43_35-CBM6	8	CBM4-GH9	1
CBM50-Peptidase	7	CBM4-GH9-Dockerin_I	1
GH23-CBM50	7	CBM41-CBM48-GH13_13	1
GH20-CBM32	6	CBM41-GH13_13	1
GH43-CBM6	6	CBM50-GH23	1
CBM20-GH77	5	CBM50-GH73	1
CBM30-GH9	5	CBM51-GH27	1
CBM48-GH13_11	5	CBM62-Dockerin_I-Cohesin_II	1
CBM48-GH13_8	5	CBM66-CBM66-GH51	1
GH2-CBM6	5	CBM68-GH13	1
GH25-CBM50	5	CBM79-CBM79-GH5_4	1
GH73-CBM50	5	CE10-GH43_35-CBM6	1
DUF3794-DUF3794-DUF3794-CBM50	5	CE12-CBM13-Dockerin_I-CBM35-CE12	1
CBM48-GH13_13	4	CE2-CBM4	1
CBM50-CBM50-GH18	4	GH5_4-CBM80-Dockerin_I	1
GH13_20-CBM34	4	Dockerin_I-CE2-CBM4	1
GH27-CBM35	4	Dockerin_I-GH16-CBM4	1
GH29-CBM32	4	GH1-CBM61	1
GH43_28-CBM32	4	GH10-CBM22	1
GH9-CBM3-Dockerin_I	4	GH10-CBM9-Dockerin_I	1
CBM50-CBM50-CBM50	4	GH11-CBM22-Dockerin_I-CBM22-CE4	1
CBM54-CBM54	3	GH116-CBM38	1
CBM32-Peptidase	3	GH128-CBM6-Dockerin_I	1
GH13_13-CBM48	3	GH13_21-CBM34	1
GH13_9-CBM48	3	GH13_7-CBM26-Dockerin_I	1
GH13-CBM48	3	GH13_8-CBM48	1
GH16-CBM4	3	GH141-CBM6-Dockerin_I	1
GH23-CBM50-CBM50	3	GH148-CBM4-GH148	1
GH31-CBM32-Dockerin_II-Cohesin_III	3	GH16-CBM13	1
DUF4959-CBM32	2	GH16-CBM16	1
SLH-SLH-SLH-CBM50	3	GH16-CBM32	1
Peptidase-CBM50	3	GH18-CBM12	1
DUF3794-DUF3794-CBM50	3	GH2-CBM32-CBM32	1
CBM13-GH64	2	GH3-CBM32	1
CBM20-CBM20-GH77	2	GH30_5-CBM62-Dockerin_I	1
CBM22-GH10	2	CBM6- CBM6- CBM6- CBM6- CBM2	1

SLH-SLH-SLH-CBM54	2	CBM62-CBM62	1
CBM22-GH10-Dockerin_I	2	CBM6-CBM6-CBM6-CBM6-CBM2	1
CBM30-GH9-CE4	2	CBM32-CBM32-CBM32-DUF4855	1
CBM34-GH13_20-GH77	2	DUF4964-CBM67-DUF5127-DUF4965-DUF1793	1
CBM35-GH26-Dockerin_I	2	GH30_8-CBM22-Dockerin-CE1	1
CBM40-GH33	2	GH35-CBM32	1
CBM66-PL11	2	GH38-CBM32	1
CBM9-CE15	2	GH43_10-CBM22-Dockerin_I-CE1	1
GH11-CBM36-CE4	2	GH43_24-GH35-CBM32	1
GH2-CBM32	2	GH43_29-CBM6-CBM22-Dockerin_I	1
GH2-CBM57	2	GH43_35-CBM36	1
GH23-CBM50-CBM50-CBM50	2	GH43_4-CBM13-Dockerin_I	1
GH25-CBM50-CBM50	2	GH9-CBM30	1
GH5_39-CBM23	2	GH9-CBM79-Dockerin_I	1
GH53-CBM61	2	GH92-CBM56	1
GH77-CBM20	2	GH97-CBM35-CBM35	1
CBM66-DUF1080-DUF1080	2	GH97-CBM35-Dockerin_I	1
DUF1735-DUF1735-CBM32	2	Peptidase-CBM32	1
DUF4959-DUF5126-CBM32	2	PL1_2-CBM37	1
DUF4959-DUF5126-CBM32-DUF5000	2	PL1_2-CBM6-Dockerin_I	1
DUF4998-CBM32	2	PL1_8-CBM13-Dockerin_I	1
CBM12-GH18-GH18	1	PL11-CBM66	1
CBM13_Dockerin_I-GH43_19-GH43_26	1	GH2-CBM51-Dockerin_I	1
CBM16-GH51	1	CBM51-CBM51	1
CBM22-CBM4-GH10	1	CBM6-CBM2	1
CBM22-Dockerin_I-CE1	1	CBM16-DUF4185	1
CBM22-GH10-CBM22-Dockerin_I-GH43	1	DUF1080-CBM66	1
CBM22-GH10-CBM22-Dockerin_I-CE1	1	DUF1080-CBM66-DUF1581	1
CBM22-GH51	1	DUF1080-DUF1080-CBM66	1
CBM22-GH51-GH43_19	1	DUF1735-CBM32	1
CBM30-CBM4-GH9	1	DUF1735-CBM40	1
CBM32-CBM32-CBM32-GH13_36	1	DUF3472-CBM32	1
CBM32-GH29	1	DUF3794-DUF3794-DUF3794-DUF3794-CBM50	1
CBM32-GH36	1	DUF4398-DUF4398-CBM50	1
CBM34-GH13_21	1	DUF4430-CBM37	1
CBM35-GH26	1	DUF4434-DUF5109-CBM32	1
CE3-CBM32-Dockerin_I	1	DUF4855-CBM32	1
DUF499-DUF1735-CBM32	1	DUF4998-CBM32-DUF5013	1
DUF5011-CBM5	1	DUF5011-CBM4	1
DUF5013-CBM61	1	DUF5011-CBM5-CBM5	1
CBM32-CBM32	1	GH44-CBM76-Dockerin_I	1
GH43-CBM32	1	GH5_39-CBM11	1
GH5_4-CBM22-Dockerin_I	1	GH5_4-CBM22-CE3-Dockerin_I	1
GH73-CBM50-CBM50	1		

Table S6 Overview of the multi-domain organizations for dockerin modules predicted in enriched rumen anaerobic consortium (ERAC)

**Domain organization**	**Number**	**Domain organization**[1]	**Number**
Dockerin_I	26	Dockerin_I-Dockerin_III	1
Peptidase-Dockerin_I	15	Dockerin_I-GH53	1
LRR_5-Dockerin_I	10	Dockerin_I-SH3	1
Dockerin_I-Peptidase	3	Dockerin_III	1
GH5-Dockerin_I	3	GH11-Dockerin_I	1
LRR-Dockerin_I	3	GH123-Dockerin_I	1
GH10-Dockerin_I	2	GH124-dockerin_I	1
GH18-Dockerin_I	2	GH127-Dockerin_I	1
LRR_5-ChW-Dockerin_I	2	GH53-Dockerin_I	1
LRR_5-LRR_5-Dockerin_I	2	GH74-dockerin_I	1
LTD-CHB-CotH-Dockerin_I	2	LRR_5-Cthe_2159-Dockerin_I	1
CE3-Dockerin_I	1	LRR_5-Dockerin_II	1
CE4-Dockerin_I	1	LRR_5-FN3-ChW-Dockerin_I	1
Peptidase-SH3-Dockerin_I	1	LRR_5-LRR_5-Tgc-Dockerin_I	1
Chitobiase-Dockerin_I	1	LRR_5-Peptidase-LRR_5-Dockerin_I	1
Cohesin_III-Cohesin_III-Dockerin_I	1	LRR-LRR-Dockerin_I	1
Cohesin_I-Dockerin_I	1	LRR-LRR-LRR-Dockerin_I	1
Dockerin_I-CE2	1	PL1-PL9-Dockerin_I	1
Dockerin_I-Cohesin_II-LRR_5-Dockerin_I	1	PL11-Dockerin_I	1
Dockerin_I-Cthe-Coth	1	Ricin_type-Dockerin_I-GH43-GH43	1
Dockerin_I-Cthe-Cthe	1	CotH-Dockerin_I-Cohesin_II	1
Calycin_like-Dockerin_I	1	Dockerin-SH3b	1
CotH-Dockerin_I	2	DUF4832-DUF4874-Dockerin_I	1
SH3-LytD-Cadherin-like-Dockerin_I	1	DUF5050-Dockerin_I	1
DUF1522-Dockerin_I	1	RiboNuclease-Cohesin_I	1
DUF285-Dockerin_I	1	RiboNuclease-Dokerin_I	1
DUF285-DUF285-Dockerin_I	1	Ricin-type-dockerin_I-GH43-GH43	1
Metallophos-CpdA-MetallophosC-Dockerin_I	1	SCP_Bacterial-Dockerin_I	1
Omp28-Dockerin_I	1	Peptidase-ChW-Dockerin_I	1

[1] – Except the domain organizations of CBM-dockerin, which are indicated in Table S5.

Table S7 Overview of the multi-domain organizations for S-layer homology domains predicted in enriched rumen anaerobic consortium (ERAC)

Domain organization[1]	Number
SLH-SLH	17
SLH-SLH-SLH	12
SLH	11
Peptidase-SLH-SLH	4
Peptidase-SLH-SLH-SLH	2
SLH-GH18	2
GH25-SLH-SLH	2
SLH-SLH-SLH-GH18	1
SLH-SLH-SLH-Cthe	1
SLH-SLH-SLH-CE-Peptidase	1
SLH-GH25	1
SLH-SLH-Amidase	1
GH25-SLH-GH25	1
GH10-SLH-SLH	1
FN3-SLH-SLH	1
DUF4430-DUF4430-SLH-SLH	1
Cthe-SLH-SLH	1
SLH-DUF3373	3
SLH-SLH_DUF5077	1

[1] – Except the domain organizations of CBM-SLH, which are indicated in Table S5.

Table S8 Multi-domain organizations for cohesin modules predicted in enriched rumen anaerobic consortium (ERAC) and their respectively location in enriched rumen anaerobic consortium genomes (ERACgs).

**Domain organization**	**ERACgs[1]**	**Number[2]**
Cohesin_I	ERACg_42; ERACg_50; ERACg_57; dataset[3]	6
Cohesin_II	ERACg_32; dataset[3]	2
Putative Cohesin	ERACg_42	3
Cohesin_I_Dockerin_I	ERACg_42	1
Dockerin_III-Cohesin_III-Dockerin_I	ERACg_42	1
Cohesin_III-Cohesin_III-Dockerin_I	ERACg_42	1
GH31-FN3-F5_F8_type_C-Dockerin_II-Cohesin_III	ERACg_37; ERACg_56	2
6 x [Cohesin_I]-CttA	ERACg_42	1

[1] Metagenome-assembly genomes (MAGs)

[2] Number of sequences coding protein with the respective domains indicated.

[3] Sequences coding proteins with cohesin modules predicted were not associated with MAGs.

Table S9. Multi-domain organization of putative cellulosomal proteins identified in *Ruminococcus* ERACg_42.

MAG ID	Predicted protein	Modular architecture	Secretion Signal[1]	Metaproteome[2]
Ruminococcus sp ERACg_42	Putative scaffoldin	6 x [Cohesin_I]-CttA	Yes	Yes
Ruminococcus sp ERACg_42	Putative scaffoldin	Cohesin I	Yes	Yes
Ruminococcus sp ERACg_42	Putative scaffoldin	Cohesin_III	Yes	Yes
Ruminococcus sp ERACg_42	Putative scaffoldin	Cohesin_I	Yes	ND[3]
Ruminococcus sp ERACg_42	Putative scaffoldin	Cohesin_I – Dockerin_I	Yes	Yes
Ruminococcus sp ERACg_42	Putative scaffoldin	Dockerin_I - Cthe_2159 - Cthe_2159	Yes	Yes
Ruminococcus sp ERACg_42	Putative scaffoldin C	without domain	No	Yes
Ruminococcus sp ERACg_42	Putative scaffoldin	without domain	Yes	Yes
Ruminococcus sp ERACg_42	Putative scaffoldin	Dockerin_III; Cohesin_III; Dockerin_I	Yes	Yes
Ruminococcus sp ERACg_42	Putative scaffoldin	Cohesin_III-Cohesin_III-Dockerin_I	Yes	ND
Ruminococcus sp ERACg_42	Putative scaffoldin	Cohesin_I	Yes	ND
Ruminococcus sp ERACg_42	Cellulosomal protein	Dockerin_I	Yes	Yes

[1] - Prediction of signal peptides is based on SignalP analysis.

[2] – Protein detected by metaproteome analysis.

[3] – No detected.

Table S10 Carbohydrate active enzymes (CAZy) encoded by *Ruminococcus* ERACg_42 genome

Enzymes	Predicted protein	Modular architecture	E.C number[1]	Secretion Signal[2]	Metaproteome[3]
Cellulases	Cellulase	GH5_1- Dockerin_I	3.2.1.4	Yes	ND[4]
	Cellulase	CBM4 - CBM30 - GH9 - Dockerin_I	3.2.1.4	Yes	ND
	Cellulase	GH5_37	3.2.1.4	No	ND
	Cellulose 1,4-beta-cellobiosidase	CBM4 - GH9	3.2.1.91	Yes	ND
	Endoglucanase	CBM79 - CBM79 - GH5_4	3.2.1.4	Yes	Yes
	Endoglucanse	GH5_1 - Dockerin_I	3.2.1.4	Yes	Yes
	Endoglucanse	GH5_1 - Dockerin_I	3.2.1.4	Yes	Yes
	Cellulase	GH9 - CBM3 - Dockerin_I	3.2.1.4	Yes	Yes
	Cellulase; acetylxylan esterase	GH5_4 - CBM22 - CE3 - Dockerin_I	3.2.1.4; 3.1.1.72	Yes	Yes
	Cellulase	GH9 - CBM3 - Dockerin_I	3.2.1.4	Yes	Yes
	Endoglucanase 1 precursor	GH9 - CBM79 - Dockerin_I	3.2.1.4	Yes	Yes
	Cellulose 1,4-beta-cellobiosidase	GH48	3.2.1.176	Yes	ND
	Cellulase	GH5_4 - CBM80 - Dockerin_I	3.2.1.4	Yes	Yes
	Cellulase	GH9 - CBM3 - Dockerin_I	3.2.1.4	Yes	Yes
	Cellulose 1,4-beta-cellobiosidase	GH9 - CBM4	3.2.1.91	Yes	Yes
	Cellulase	GH9 - CBM3 - Dockerin_I	3.2.1.4	Yes	Yes
	Glycoside hydrolase family 44	GH44 - CBM76 – Dockerin_I	---	Yes	Yes
Endo-hemicellulases	Endo-1,4-beta-xylanase	CBM22 – GH10 - Dockerin_I	3.2.1.8	Yes	ND
	Xyloglucan-specific endo-beta-1,4-glucanase	GH5_4 - CBM22 – Dockerin_I	3.2.1.151	Yes	Yes
	Putative glycoside hydrolase family 130	GH130	----	No	ND
	Endo-β-1,4-xylanase	GH11 - Dockerin_I	3.2.1.8	Yes	ND

Endo-β-1,4-xylanase; Chitin deacetylase	GH11 – CBM22 - Dockerin_I – CBM22 – CE4	3.2.1.8; 3.5.1.41	Yes	ND
Arabinogalactan endo-beta-1,4-galactanase	GH53 - Dockerin_I	3.2.1.89	Yes	ND
Endo-1,3-beta-glactosidase	GH16 – CBM4	3.2.1.103	Yes	ND
Putative mannan endo-1,4-beta-mannosidase	CBM35 - GH26- Dockerin_I	3.2.1.78	Yes	ND
Putative mannan endo-1,4-beta-mannosidase	CBM35 - GH26	3.2.1.78	Yes	ND
Galactan endo-1,6-beta-galactosidase	GH30_5- CBM62 - Dockerin_I	3.2.1.164	Yes	ND
Putative glycoside hydrolase family 141	GH141- CBM6 - Dockerin_I	---	Yes	ND
Oligoxyloglucan reducing-end-specific cellobiohydrolase	GH74 – Dockerin_I	3.2.1.150	Yes	Yes
Mannan endo-1,4-beta-mannosidase	CBM35 – GH26 – Dockerin_I	3.2.1.78	Yes	Yes
Endo-1,4-beta-xylanase; feruloyl esterase	GH10 - CBM22 – CE1	3.2.1.8; 3.1.1.73	Yes	Yes
Endo-1,4-beta-xylanase; Non-reducing end alpha-L-arabinofuranosidase	CBM22 - GH10 - CBM22 – Dockerin_I – GH43 - CBM36	3.2.1.8; 3.2.1.55	Yes	Yes
Endo-1,4-beta-xylanase	CBM22 – GH10 – Dockerin_I	3.2.1.8	Yes	Yes
Glucuronoarabinoxylan endo-1,4-beta-xylanase; feruloyl esterase	GH30_8 - CBM22 - Dockerin_I – CE1	3.2.1.136; 3.1.1.73	Yes	Yes
Endo-1,4-beta-xylanase; feruloyl esterase	GH43_10 - CBM22 – Dockerin_I – CE1	3.2.1.37 3.1.1.73	Yes	Yes
Xylan 1,4-beta-xylosidase	GH43_29 - CBM6 - CBM22 - Dockerin_I	3.2.1.37	Yes	Yes
Arabinan endo-1,5-alpha-L-arabinosidase	GH43 – CBM13 - Dockerin_I	3.2.1.99	Yes	ND
Putative Carbohydrate esterase family 1	CE1	----	No	ND
Putative Carbohydrate esterase family 1	CE1	----	No	ND
Feruloyl esterase	CBM22-CE1	3.1.1.73	Yes	ND
Putative Carbohydrate esterase family 1	CE1	-------	Yes	ND
Putative Carbohydrate esterase family 1	CE1	-------	Yes	ND
Putative Carbohydrate esterase family 1	CE1	-------	Yes	ND
Putative acetylxylan esterase	CE2-CBM4	3.1.1.72	Yes	ND
Acetylxylan esterase	CE2-Dockerin_I	3.1.1.72	Yes	ND
Putative Carbohydrate esterase family 3	CE3	-------	Yes	ND
Putative Carbohydrate esterase family 3	CE3-Dockerin_I	-------	Yes	ND

	Putative Carbohydrate esterase family 3	CE3	-------	Yes	ND
	Putative Carbohydrate esterase family 3	CE3	-------	Yes	ND
	Putative chitin deacetylase	CE4	3.5.1.41	Yes	ND
	Putative Carbohydrate esterase family 4	CE4-Dockerin_I	-------	Yes	ND
	Putative glycoside hydrolase family 127	GH127 - Dockerin_I	-----	Yes	ND
Amylases	1,4 –alpha-glucan branching	CBM48 - GH13_9	2.4.1.18	No	ND
	1,4 –alpha-glucan branching	CBM48 - GH13_9	2.4.1.18	No	ND
	Cyclomaltodextrinase	GH13_20	3.2.1.54	No	ND
	Cyclomaltodextrinase	GH13_20	3.2.1.54	No	ND
	α-amylase	GH13_20	3.2.1.1	No	ND
	4- alpha-glucanotransferase	GH77	2.4.1.25	No	ND
Oligosaccharidases	Beta-galactosidase	GH2	3.2.1.23	No	ND
	Putative beta-glucosidase	GH3	3.2.1.21	Yes	ND
	Putative beta-glucosidase	GH3	3.2.1.21	No	ND
	α -D-xyloside xylohydrolase	GH31	3.2.1.177	No	ND
	β-galactosidase	GH42	3.2.1.23	No	ND
	α -l-fucosidase	GH95	3.2.1.51	No	ND
	β-galactosidase	GH2 - Dockerin_I	3.2.1.23	Yes	Yes
	non-reducing end alpha-L-arabinofuranosidase	GH43 – CBM6	3.2.1.55	No	ND
	non-reducing end alpha-L-arabinofuranosidase	Dockerin_I - GH43_26	3.2.1.55	Yes	ND
Chitinase	Chitinase	GH18 - Dockerin_I	3.2.1.14	Yes	ND
	Putative Chitinase	GH18 - Dockerin_I	3.2.1.14	Yes	ND
Pectinase	Putative rhamnogalacturonyl hydrolase	GH105	3.2.1.172	No	ND
	Carbohydrate esterase family 12	CE12-CBM13-Dockerin_I-CBM35-CE12	3.1.1.86	Yes	Yes

	Putative Carbohydrate esterase family 2	CE12-Dockerin_III	-------	Yes	ND
	Rhamnogalacturonan endolyase	PL11 - Dockerin_I	4.2.2.23	Yes	Yes
	Pectate lyase	PL1 – PL9 – Dockerin_I	4.2.2.2; 4.2.2.9	Yes	Yes
Lysozyme	Lysozyme	GH25	3.2.1.17	Yes	ND
	Lysozyme	GH25	3.2.1.17	Yes	ND
	Lysozyme	GH25	3.2.1.17	No	ND
	Putative lysozyme	GH25	3.2.1.17	No	ND
	Lysozyme	GH25	3.2.1.17	No	ND
Licheninase	Putative licheninase	Dockerin_I - GH16 – CBM4	3.2.1.73	Yes	ND

[1] - Enzyme Commission number

[2] - Prediction of signal peptides is based on SignalP analysis.

[3] - Protein detected by metaproteome analysis.

[4] - No detected.

Table S11. Identity of cellulosomal proteins shared with sequences available in NCBI non-redundant Protein Database.

**Predicted protein**	**Modular architecture**	**Query Cover (%)**	**Identity (%)[1]**	**Accesssion[2]**
Putative scaffoldin	6x [Cohesin_I]-CttA	67	49.74	CAO00834.2
Putative scaffoldin	Cohesin	15	30.66	CAC34385.1
Putative scaffoldin	Cohesin_III	48	36.26	CAH18996.1
Putative scaffoldin	Cohesin_I	93	42.29	CAO02529.1
Putative scaffoldin	Cohesin_I – Dockerin_I	100	85.16	AEV59152.1
Cellulosomal protein	Dockerin_I - Cthe_2159 - Cthe_2159[3]	Not found	Not found	Not found
Putative scaffoldin	*without domain*	94	76.9	AEV59152.1
Putative scaffoldin	Dockerin_III; Cohesin_III; Dockerin_I	48	32.76	WP_021680552.1
Leucine-rich repeat	LRR_5 – Dockerin_I	55	31.21	WP_080550469.1
Leucine rich repeats	LRR_5 – Dockerin_I	29	37.63	SFY02195.1
Leucine rich repeats	LRR_5 –LRR_5 – Dockerin_I	89	53.89	WP_019678006.1
Leucine rich repeats	LRR_5 – Dockerin_I	82	30.59	WP_028517173.1

[1] Best Blast hit reference protein sequences from NCBI database (non-redundant Protein Database)

[2] GenBank accession number.

[3] Sequence predicted based on Pfam database.

Table S12. CAZy profile of the enriched rumen anaerobic consortium (ERAC) and selected previous studies

			Total Number										
**Consortium/Inoculum**	**Enrichment substrate**	**Assembled DNA (Mbp)**	**Contigs**	**CDSs[1]**	**CAZymes[2]**	**GHs**	**CBMs**	**CEs**	**PLs**	**AAs**	**GTs**	**GHs/Mb**	**Refs.**
Anaerobic condition[3]													
**ERAC/Cow rumen**	**Bagasse**	**197**	**103,541**	**142,703**	**4,974**	**2,158**	**697**	**858**	**69**	**176**	**1,462**	**10.9**	**This study**
BD-C/Beaver droppings	Cellulose	78	5,010	71,348	3,093	1,176	440	191	88	0	1,198	15	(4)
BD-PH/ Beaver droppings	Poplar hydrolysate	81	10,558	81,969	2,982	1,371	429	219	24	0	939	16.9	(4)
MR-C/Moose rumen	Cellulose	58	5,705	56,127	1,907	667	278	97	17	0	848	11.5	(4)
MR-PH/Moose rumen	Poplar hydrolysate	67	6,941	66,970	2,612	1,047	509	188	23	0	843	15.6	(4)
Aerobic condition[4]													
APACMC/Cattle manure	Apple pomace	182	272,516	220,767	9,274	3,245	1,235	1,621	199	430	2,494	17.8	(12)
ZCTH02/Composting	CMC[5]	20.6	1,468	19,561	691	225	120	137	5	32	162	10.9	(11)
EMSD5/Composting	Corn stover	39	17,908	48,263	1,537	691	392	270	41	56	348	17.7	(10)

1 – Protein coding sequences.

2 – Total CAZymes predicted.

3 – Only the enriched cultures under restrict anaerobic condition were considered.

4 – Enriched cultures under static conditions

5- Carboxymethyl cellulose.

Abbreviations: CAZymes, Carbohydrate-active enzyme; GH, glycoside hydrolase; CBM, carbohydrate-binding module; CE, carbohydrate esterases; PL, polysaccharide lyases; AA, auxiliary activities; GT, glycosyltransferases.

Table S13 The number of different CAZyme families identified in the enriched rumen anaerobic consortium (ERAC) and selected previous studies.

Consortium/Inoculum	Enrichment substrate	CDSs[1]	Total CAZymes[2]	Number of Different CAZy families					GHs/Mb	Refs.
				GHs	CBMs	CEs	PLs	AAs		
Anaerobic condition[3]										
**ERAC/Cow rumen**	**Bagasse**	**142,703**	**4,974**	**92**	**43**	**14**	**12**	**5**	**10.9**	**This study**
BD-C/Beaver droppings	Cellulose	71,348	3,093	87	31	12	13	0	15	(4)
BD-PH/ Beaver droppings	Poplar hydrolysate	81,969	2,982	84	27	13	7	0	16.9	(4)
MR-C/Moose rumen	Cellulose	56,127	1,907	74	28	12	6	0	11.5	(4)
MR-PH/Moose rumen	Poplar hydrolysate	66,970	2,612	79	32	12	7	0	15.6	(4)
Aerobic condition[4]										
APACMC/Cattle manure	Apple pomace	220,767	9,274	94	47	16	15	7	17.8	(12)
ZCTH02/Composting	CMC[5]	19,561	691	46	16	12	4	5	10.9	(11)
EMSD5/Composting	Corn stover	48,263	1,537	75	36	12	11	5	17.7	(10)

[1] – Protein coding sequences.
[2] – Total CAZymes predicted.
[3] – Only the enriched cultures under restrict anaerobic condition were considered.
[4] – Enriched cultures under static conditions
[5]- Carboxymethyl cellulose.
Abbreviations: CAZymes, Carbohydrate-active enzyme; GH, glycoside hydrolase; CBM, carbohydrate-binding module; CE, carbohydrate esterases; PL, polysaccharide lyases; AA, auxiliary activities; GT, glycosyltransferases.

Table S14. Chemical composition of the sugarcane bagasse sample used as carbon source by enriched rumen anaerobic consortium.

	Bagasse Composition (%)				
	Cellulose	Hemicellulose	Lignin	Ash	Extractives
Bagasse sample[1]	79,78 ± 0,40	2,29 ± 0,12	9,53 ± 0,51	6,23 ± 0,21	ND ± ND

[1] Bagasse was treatment as followed: hydrothermal processing (190 °C for 10 min) and delignificated with NaOH 1.0% (w/v), 100 °C for 1 h (13).
An average (± standard deviation) of triplicate determinations.
ND, no data available.

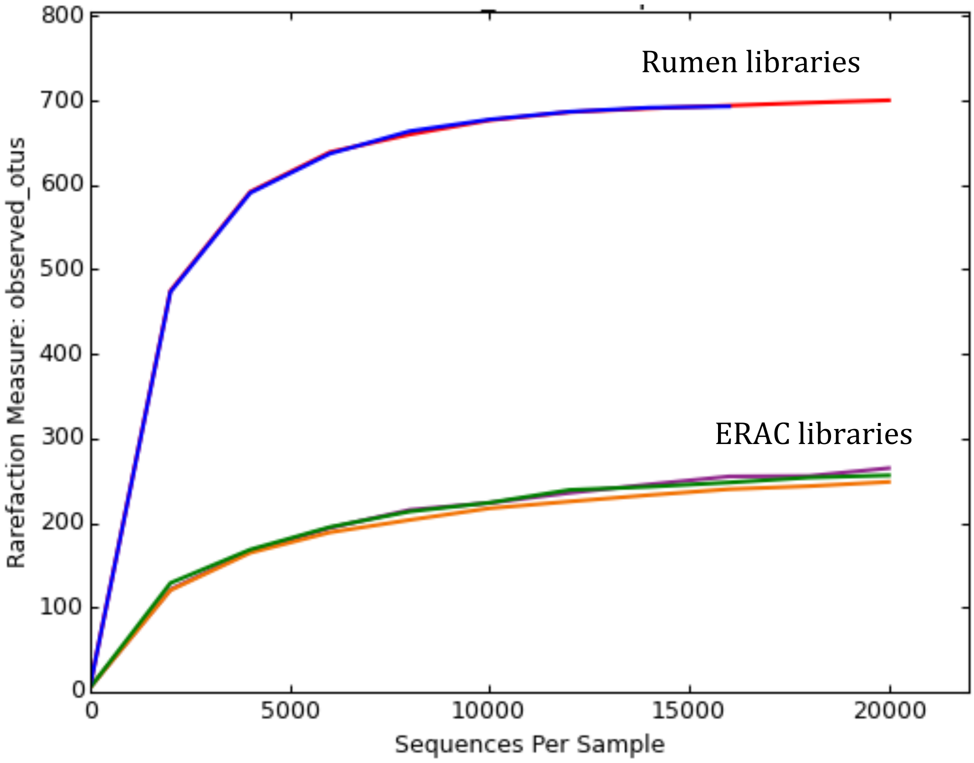

Fig. S1. Rarefaction curves of 16S rRNA amplicons from enriched rumen anaerobic consortium (ERAC) and rumen sample. The rarefaction curves at a cutoff level of 3% of each replicate are shown in different color.

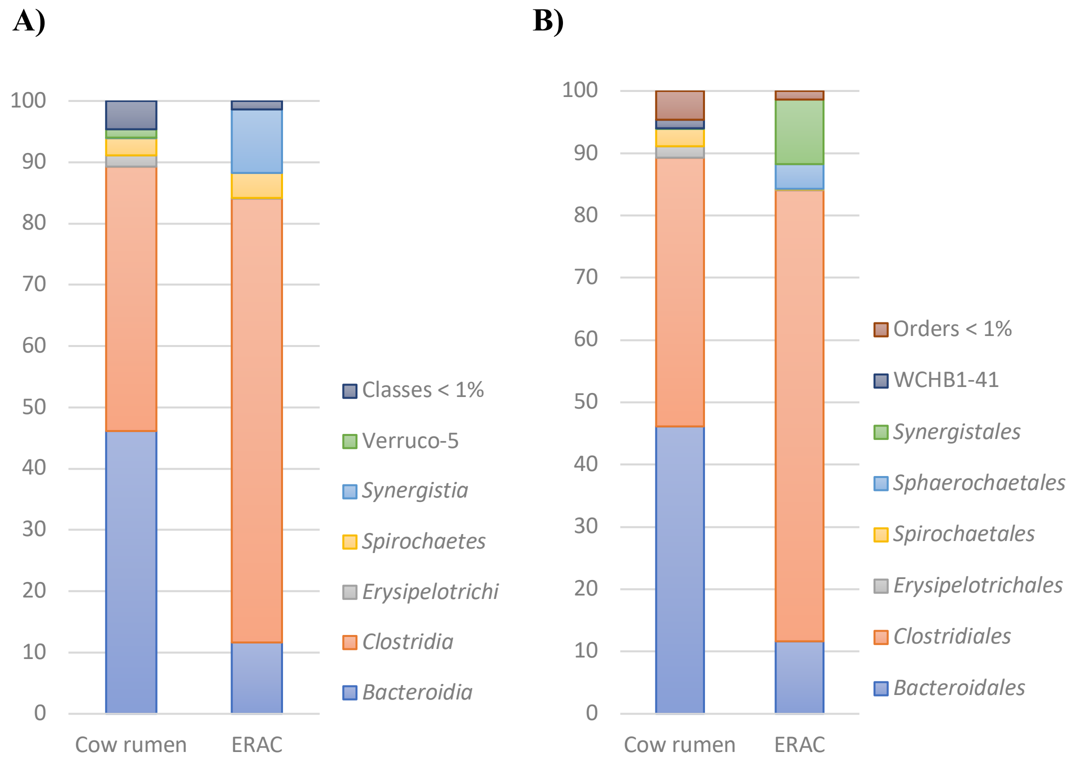

Fig. S2. Relative abundance of the classes (A) and order (B) ranks present in the cow rumen sample and enriched rumen anaerobic consortium (ERAC). Abundances were determined based on 16S rRNA gene amplicon sequences. Classes and orders represented by less than 1 % of the total reads were combined in the group named "Classes" and "Orders", respectively.

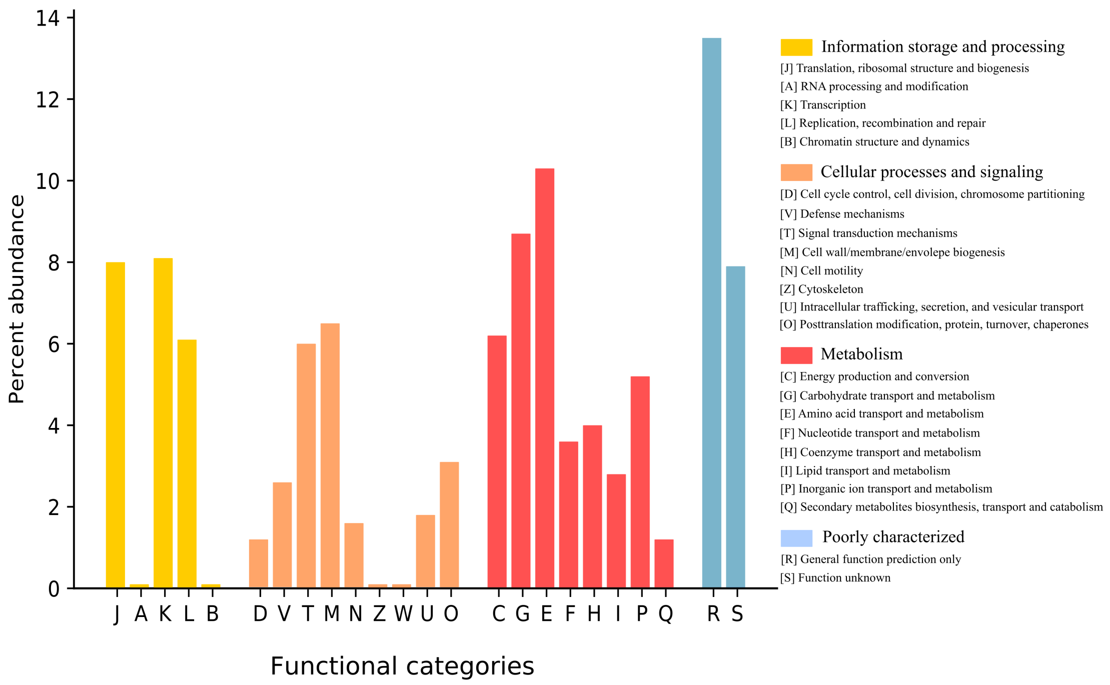

Fig. S3. The relative abundance of the annotated proteins from ERAC metagenome data according COG categories.

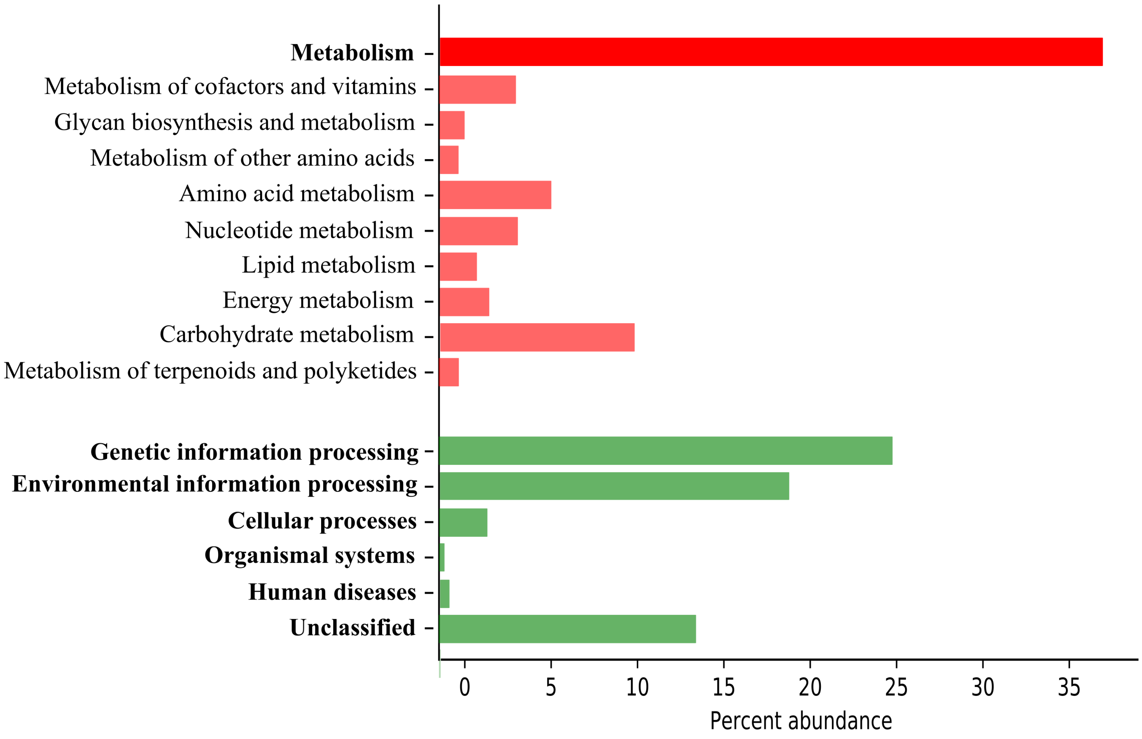

Fig. S4. The relative abundance of the annotated proteins from ERAC metagenome data according KEGG database.

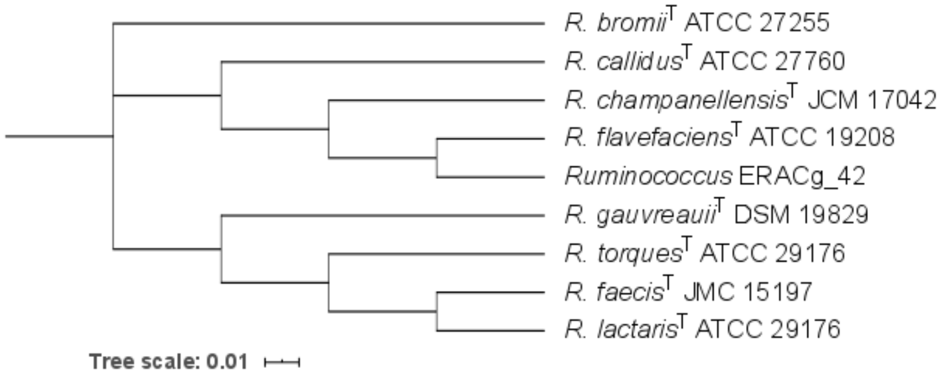

Fig. S5. Phylogenomic tree based on the concatenation of 304 orthologous proteins showing the relationships between *Ruminococcus* ERACg_42 and type strains of *Ruminococcus* strains. The tree was constructed within the EDGAR platform (14) using standard settings. Bar, 0.01 nt changes per position.

References

1. Mai-Gisondi G, Maaheimo H, Chong SL, Hinz S, Tenkanen M, Master E. 2017. Functional comparison of versatile carbohydrate esterases from families CE1, CE6 and CE16 on acetyl-4-O-methylglucuronoxylan and acetyl-galactoglucomannan. Biochim Biophys Acta - Gen Subj 1861:2398–2405.
2. Aragunde H, Biarnés X, Planas A. 2018. Substrate recognition and specificity of chitin deacetylases and related family 4 carbohydrate esterases. Int J Mol Sci 19:1–30.
3. Biely P, Malovíková A, Uhliariková I, Li XL, Wong DWS. 2015. Glucuronoyl esterases are active on the polymeric substrate methyl esterified glucuronoxylan. FEBS Lett 589:2334–2339.
4. Wong MT, Wang W, Couturier M, Razeq FM, Lombard V, Lapebie P, Edwards EA, Terrapon N, Henrissat B, Master ER. 2017. Comparative metagenomics of cellulose- and poplar hydrolysate-degrading microcosms from gut microflora of the Canadian Beaver (Castor canadensis) and North American moose (Alces americanus) after long-term enrichment. Front Microbiol 8:1–14.
5. Gharechahi J, Salekdeh GH. 2018. A metagenomic analysis of the camel rumen's microbiome identifies the major microbes responsible for lignocellulose degradation and fermentation. Biotechnol Biofuels 11:1–19.
6. Ransom-Jones E, McCarthy AJ, Haldenby S, Doonan J, McDonand JE. 2017. Lignocellulose-degrading microbial communities in landfill sites represent a repository of unexplored biomass-degrading diversity. mSphere 2:1–13.
7. Cantarel BI, Coutinho PM, Rancurel C, Bernard T, Lombard V, Henrissat B. 2009. The Carbohydrate-Active EnZymes database (CAZy): An expert resource for glycogenomics. Nucleic Acids Res 37:233–238.
8. Ohnuma T, Onaga S, Murata K, Fukamizo T, Taira T, Katoh E. 2009. Structure and Function of Family 50 Carbohydrate Binding Modules (LysM Domains) from Pteris ryukyuensis Chitinase-A. J Appl Glycosci 56:97–104.
9. Svartström O, Alneberg J, Terrapon N, Lombard V, de Bruijn I, Malmsten J, Dalin A-M, EL Muller E, Shah P, Wilmes P, Henrissat B, Aspeborg H, Andersson AF. 2017. Ninety-nine de novo assembled genomes from the moose (Alces alces) rumen microbiome provide new insights into microbial plant biomass degradation. ISME J 1–14.

10. Zhu N, Yang J, Ji L, Liu J, Yang Y, Yuan H. 2016. Metagenomic and metaproteomic analyses of a corn stover-adapted microbial consortium EMSD5 reveal its taxonomic and enzymatic basis for degrading lignocellulose. Biotechnol Biofuels 9:243.
11. Lemos LN, Pereira R V., Quaggio RB, Martins LF, Moura LMS, da Silva AR, Antunes LP, da Silva AM, Setubal JC. 2017. Genome-centric analysis of a thermophilic and cellulolytic bacterial consortium derived from composting. Front Microbiol 8.
12. Zhou M, Guo P, Wang T, Gao L, Yin H, Cai C, Gu J, Lü X. 2017. Metagenomic mining pectinolytic microbes and enzymes from an apple pomace-adapted compost microbial community. Biotechnol Biofuels 10:1–15.
13. Gouveia ER, Nascimento RT do, Souto-Maior AM, Rocha GJ de M. 2009. Validação de metodologia para a caracterização química de bagaço de cana-de-açúcar. Quim Nova 32:1500–1503.
14. Blom J, Albaum SP, Doppmeier D, Pühler A, Vorhölter F-J, Zakrzewski M, Goesmann A. 2009. EDGAR: a software framework for the comparative analysis of prokaryotic genomes. BMC Bioinformatics 10:154.
